# Retreatment after loss of follow-up in adolescent and young adult patients living with HIV: a case-control study^*^

**DOI:** 10.1590/1518-8345.7973.4801

**Published:** 2026-06-15

**Authors:** Camila Moraes Garollo Piran, Alana Vitória Escritori Cargnin, Mariana Martire Mori, Rosana Rosseto de Oliveira, João Manuel Graça Frade, Marcela Demitto Furtado

**Affiliations:** 1 Universidade Estadual de Maringá, Maringá, PR, Brazil.; 2 Polytechnic University of Leiria, Leiria, Portugal.; 3 Scholarship holder at the Coordenação de Aperfeiçoamento de Pessoal de Nível Superior (CAPES), Brazil.

**Keywords:** Retreatment, Lost to Follow-Up, Adolescents, Young Adult, HIV, Acquired Immunodeficiency Syndrome

## Abstract

**Objective:**

to identify factors associated with retreatment after loss to follow-up among adolescents and young adults living with Human Immunodeficiency Virus.

**Method:**

paired case-control study (1:1 ratio) with data collection from medical records. The cases were adolescents and young adults diagnosed with Human Immunodeficiency Virus/Acquired Immunodeficiency Syndrome who underwent retreatment after loss to follow-up. The controls were adolescents and young adults diagnosed with the disease who were lost to follow-up and had no history of retreatment. Logistic regression was used for statistical analysis.

**Results:**

76 cases and 76 controls were included. The variable associated with the highest chance of retreatment was not having a religion (ORaj: 3.46; 95% CI: 1.52-7.88; p=0.003). The factors that decreased the chances of retreatment were 21.91 years of age at the beginning of follow-up (ORaj: 0.78; 95% CI: 0.62-0.98; p=0.039), female gender (ORaj: 0.12; 95% CI: 0.03-0.45; p=0.001), living in an institution (ORaj: 0.72; 95% CI: 0.12-0.43; p=0.004), alcohol use (ORaj: 0.20; 95% CI: 0.08-0.49; p<0.001), diagnosis of Human Immunodeficiency Virus at the Treatment and Counseling Center (ORaj: 0.31; 95% CI: 0.13-0.74; p=0.008), and missed appointment(s) before loss to follow-up (ORaj: 0.20; 95% CI: 0.07-0.52; p=0.001).

**Conclusion:**

the return to treatment of adolescents and young people living with Human Immunodeficiency Virus is multifactorially determined by sociodemographic, behavioral, and clinical profiles.

## Highlights

**(1)** First Brazilian study addressing re-treatment among young people living with HIV. **(2)** Lack of religious affiliation was associated with higher odds of retreatment. **(3)** Sex, alcohol use, and living in an institution are associated with re-treatment. **(4)** Diagnosis at the TCC and missed appointments decreased the chances of re-treatment. **(5)** Provides insights into the prevention of permanent dropout.

## Introduction

It is estimated that in 2023, 40 million people were living with Human Immunodeficiency Virus (HIV), with two out of every seven new HIV infections occurring among adolescents and young people^1^. Even though antiretroviral therapy (ART) has had a significant impact on reducing morbidity and mortality among PLHIV, adolescents and young people are more exposed to negative contexts^2^, as this age group is often absent from the HIV treatment cascade^3^.

The introduction of ART has made HIV a chronic, manageable health condition, providing a better quality of life, a return to normal life expectancy, and substantial impacts on HIV prevention and treatment. However, for adolescents and young people living with this condition, it is still challenging to maintain adherence to treatment due to the daily use of ART throughout their lives^4^ ^-^ ^6^.

Continued non-adherence or loss to follow-up causes lengthy interruptions in treatment or discontinuation of ART, which is one of the main barriers to ending AIDS (Acquired Immunodeficiency Syndrome), as it can lead to drug resistance and, consequently, treatment failure^5^ ^-^ ^6^. In addition, adolescents and young people living with HIV have worse outcomes in relation to HIV treatment when compared to other age groups living with HIV, due to the health risk behaviors they exhibit in this age group^5^.

That said, many adolescents and young people living with HIV abandon treatment^7^ ^)^ and return to the service after a specific period^8^. Returning to HIV care after loss to follow-up has become a common, yet poorly understood, behavior but one that is still poorly understood. Retreatment is increasingly present in the HIV care cascade, in which many individuals enter and leave care after starting treatment and throughout their lives^8^ ^-^ ^9^.

Although the factors that drive loss to follow-up or abandonment are studied^7^ ^,^ ^10^ ^-^ ^11^, little is known about the factors that facilitate and hinder return to treatment^8^ ^-^ ^9^. The results for this specific population are still incipient and, consequently, make it impossible for health professionals, such as nurses involved in service management, to identify the factors associated with retreatment and to implement preventive strategies to avoid permanent discontinuation^8^.

Exploring re-treatment after loss to follow-up in a setting that provides treatment in different contexts may contribute to the implementation of new strategies to improve outcomes among adolescents and young people, which will be crucial for meeting the global targets for AIDS elimination by 2030. Thus, this study aims to answer the following research question: What factors are associated with returning to HIV care among adolescents and young people? That said, the objective of this study was to identify the aspects related to retreatment after loss to follow-up among adolescents and young people living with HIV.

## Method

### Type of study

Epidemiological study, case-control type, paired with a ratio of 1 case/1 control of adolescents and young people living with HIV, which was nested in an ambispective cohort. The 1:1 ratio was established based on the time available for conducting this research. The Strengthening the Reporting of Observational Studies in Epidemiology (STROBE) was used to report the study, as recommended by the Enhancing the Quality and Transparency of Health Research (EQUATOR Network)^12^.

### Study location

The study was conducted at the Specialized Care Service (SAE) belonging to the Sexually Transmitted Infections/HIV/AIDS Outpatient Clinic of the 15th Regional Health Department, located in the northwest of the state of Paraná, Brazil. It is a specialized public referral service that provides care to Maringá and 29 other municipalities.

### Population

Adolescents and young adults diagnosed with HIV/AIDS according to the International Classification of Diseases (ICD-10)-an indicator of mortality and morbidity statistics-represented by codes B20.0 to B24, with subsequent retreatment after loss to follow-up, were defined as cases. Meanwhile, the controls were adolescents and young people with ICD-10 related to HIV/AIDS lost to follow-up in the SAE after starting ART, with no history of retreatment.

The following inclusion criteria were considered: being between 10 and 24 years of age, having acquired HIV through sexual transmission, and having started ART. It should be noted that the age groups 10 to 19 years and 20 to 24 years correspond to adolescence and youth, respectively^13^. The following were defined as non-inclusion criteria: patients who did not start ART and those who acquired HIV through vertical transmission. The exclusion criteria were patients who died and cases or controls without matching.

### Sample definition

A total sample of 198 adolescents and young people living with HIV who were in a situation of loss to follow-up and were treated at the service between January 2017 and December 2023 was used. After an exploratory analysis of the medical records of adolescent and young patients that verified the above selection criteria, sample pairing was performed to identify the most significant possible similarity between individuals.

**Figure 1:**
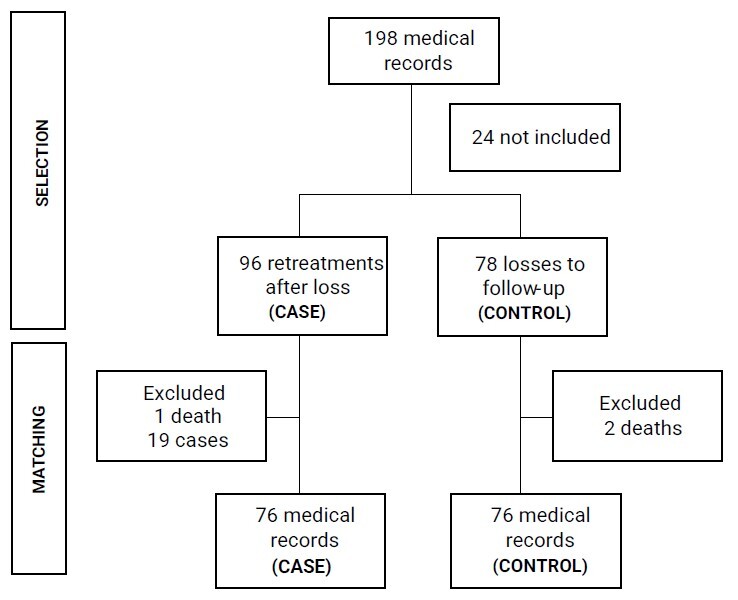
Selection of the medical records of adolescents and young people living with HIV. Maringá, PR, Brazil, 2024

The groups were matched by age group and year of entry into the service. The age group variable was included in the pairing to provide greater similarity in age between cases and controls; even though some pairs did not match exactly, they were still close. Nineteen patients who did not start ART and five patients who acquired HIV through vertical transmission were not included. Three deaths and 19 patients who were cases were excluded due to the lack of control within the study period (Figure 1).

### Study variables

Patient information regarding each variable was collected from medical records. The dependent variable (retreatment after loss to follow-up) was used in logistic regression to assess the association with the independent variables.

*Dependent variable*

Retreatment after loss to follow-up was adopted as the dependent variable (outcome). Retreatment or return to care is defined as treatment of the patient after abandonment, with repetition of the initial treatment, or with an additional or alternative measure. In situations where the original modality was abused, detrimental, or ineffective, retreatment is frequently used in reference to another one^8^ ^-^ ^9^ ^,^ ^14^.

*Independent variables*

The following characteristics were grouped as independent variables:

- Sociodemographic: age at diagnosis (in years), age at the start of follow-up (in years), sex (male; female), marital status (single; with partner), race/color (white; non-white), education (<12 years; ≥12 years), religion (no; yes), sexual orientation (heterosexual; homosexual/bisexual), occupation (employed/self-employed; student/homemaker; unemployed), housing (with family; with friends; in an institution; alone; unknown);
- Behavioral: alcohol use (no; yes), tobacco use (no; yes), drug use (no; yes), sexual partnership (steady; casual), comorbidity(ies) (no; yes), mental disorder(s) (no; yes);
- Clinical, immunological, and laboratory: diagnosis at the Counseling and Testing Center (CTC) (no; yes), type of admission (new case; transferred case), time to initiation of ART after diagnosis (in days), initial ART regimen [Lamivudine (3TC) + Tenofovir (TDF) + Dolutegravir (DTG); Lamivudine (3TC) + Tenofovir (TDF) + Efavirenz (EFZ); Other], ART side effect (no; yes), number of regimens used (1; more than 1), HIV staging according to WHO (stage I; stage II; stage III; stage IV; unknown), first viral load (VL) result (suppressed; not suppressed; undetectable; unknown), first CD4+ T lymphocyte count (CD4+) (in cells/mm³), VL result before loss to follow-up (suppressed; not suppressed; undetectable; unknown) CD4+ count before loss to follow-up (in cells/mm^3^), opportunistic infection(s) (no; yes; unknown), other sexually transmitted infection(s) (no; yes; unknown), missed appointment(s) before loss to follow-up (no; yes).

### Instruments used to collect information

Data were collected from secondary sources, based on health records made available at the Sexually Transmitted Infections/HIV/AIDS outpatient clinic. The data collection instrument was established as recommended by the Ministry of Health, in accordance with the notification and outpatient follow-up form for HIV cases. To ensure data comparability, the same instrument was used for cases and controls.

### Data collection

The data were collected between June and October 2024, with the participation of two research assistants, namely, one master’s student and one doctoral student, both participants in the research project. The research assistants were trained on-site by the principal investigator and assisted in collecting and double-checking data to ensure the accuracy of the information. Both were blinded to the research objectives and the study question as a strategy to minimize confounding bias in the possibility of establishing any relationship between the responses. The information was entered into and stored in a database managed by the principal investigator. In addition to pairing, the registration variable and the medical record number variable were used to differentiate medical records better and minimize selection bias.

### Data processing and analysis

Microsoft Excel^®^ was used for data processing, and the data were then transferred to SPSS Statistics^®^ version 25.0 for analysis. First, the sociodemographic, behavioral, clinical, immunological, and laboratory characteristics of adolescents and young adults living with HIV were described according to retreatment status after loss to follow-up, using measures of absolute and relative frequency, central tendency (mean), and dispersion (standard deviation). Next, binomial logistic regression models were used to verify the factors associated with re-entry into treatment among adolescents and young adults who had discontinued treatment.

Bivariate analyses were performed to compare each variable with the outcome under study. Those with p-values ≤ 0.25 in the Wald chi-square test were evaluated for multicollinearity to construct parsimonious, robust multivariate models. Multiple models were then tested using stepwise backward variable selection. Independent variables with p-values < 0.05 in this test were retained in the final models after adjustment for one another. 

As measures of association, odds ratios (OR) and adjusted odds ratios (ORaj) were calculated, along with their 95% confidence intervals (95% CI). These ratios represented the increased or decreased likelihood of each sub-variable for re-treatment after loss to follow-up, relative to the reference subcategories in the respective independent variables. The final model was found to be significant relative to the null in the omnibus test.

For the final multiple models, a receiver operating characteristic (ROC) analysis was performed, a valuable method for evaluating the model’s predictive accuracy. From this, the area under the curve (AUC) was calculated, representing the probability that the prediction is in the correct order when a test variable is observed. In addition, the sensitivity and specificity of the final model were calculated at the optimal cutoff point, defined by the Youden index.

### Ethical aspects

Even though there was no direct contact with the participants, the medical records contained confidential information. Thus, in accordance with Resolutions No. 466/12 and 510/16 of the National Health Council (CNS), the study was submitted to the Permanent Committee on Ethics in Research Involving Human Beings of the signatory institution and was approved under opinion No. 5,202,623/2022 and No. 6,897. 755/2024, Certificate of Ethical Review (CAAE): 52331221.3.0000.0104.

## Results

A total of 198 medical records of adolescents and young adults living with HIV were analyzed, with 152 eligible for the study, corresponding to 76 cases (retreatment) and 76 controls (non-retreatment). Regarding the sociodemographic and behavioral characteristics of adolescents and young adults in the cases and controls, the mean age at diagnosis was 21.39 years and 21.65 years, respectively. In both groups, the majority were male and single.

A statistically significant association was identified between retreatment and the variables age at the start of follow-up (p=0.062), gender (p=0.010), race/color (p=0.233), religion (p=0.003), occupation (p=0.114), housing (p=0.032), and alcohol use (p=0.022). The chance of retreatment after loss to follow-up was higher among individuals of non-white race/color (OR: 1.55; 95% CI: 0.75-3.18), those without religion (OR: 2.70; 95% CI: 1.39-5.25), who are unemployed (OR: 3.04; 95% CI: 0.76-12.12), and who live in an institution (OR: 0.17; 95% CI: 0.03-0.86) (Table 1).

**Table 1:** Descriptive and bivariate analysis of sociodemographic and behavioral characteristics of adolescents and young people living with HIV, according to the outcome of retreatment or not after loss to follow-up. Maringá, PR, Brazil, 2024

Variable	Case	Control	Bivariate analysis (crude)		
	n(%)	n(%)	OR*	CI95%^†^	p-value^‡^
**Age at diagnosis**					
Continuous variable (in years)	21.39(1.96)^§^	21.65(2.00)^§^	1.05	0.89-1.25	0.413
**Age at start of follow-up**					
Continuous variable (in years)	21.91(1.84)^§^	22.47(1.79)^§^	0.84	0.98-1.42	**0.062**
**Gender**					
Male	56(73.68)	69(90.79)	Reference		
Female	20(26.32)	7(15.79)	0.29	0.11-0.74	**0.010**
**Marital status**					
Without a partner	60(78.95)	65(85.53)	Reference		
With partner	16(21.05)	11(14.47)	0.65	0.28-1.52	0.326
**Race/color**					
White	58(76.32)	51(67.11)	Reference		
Not white	18(23.68)	25(32.89)	1.55	0.75-3.18	**0.233**
**Education**					
<12 years	26(34.21)	24(31.58)	Reference		
≥12 years	50(65.79)	52(68.42)	1.08	0.54-2.14	0.818
**Religion**					
No	29(38.16)	48(63.16)	2.70	1.39-5.25	**0.003**
Yes	47(61.84)	28(36.84)	Reference		
**Sexual orientation**					
Heterosexual	23(30.26)	17(22.37)	Reference		
Homosexual/bisexual	53(69.74)	59(77.63)	1.45	0.70-3.02	0.313
**Occupation**					
Employed/self-employed	57(75.00)	50(65.79)	Reference		
Student/homemaker	16(21.05)	18(23.68)	1.14	0.51-2.52	0.741
Unemployed	3(3.95)	8(10.53)	3.04	0.76-12.12	**0.114**
**Housing^||^**					
With family members	49(64.47)	36(10.53)	Reference		
With friends	2(2.63)	12(15.79)	1.42	0.69-2.90	0.337
In an institution	2(2.63)	3(3.95)	0.17	0.03-0.86	**0.032**
Alone	23(30.26)	24(31.58)	0.69	0.01-4.55	0.705
Ignored	0(0.00)	1(1.32)			
**Alcohol use**					
No	25(32.89)	39(51.32)	Reference		
Yes	51(67.11)	37(48.68)	0.46	0.23-0.89	**0.022**
**Tobacco use**					
No	46(60.53)	47(61.84)	Reference		
Yes	30(39.47)	29(38.16)	0.88	0.45-1.70	0.706
**Drug use**					
No	52(68.42)	54(71.05)	Reference		
Yes	24(31.58)	22(28.95)	0.85	0.42-1.73	0.669
**Sexual partnership**					
Fixed	39(51.32)	34(44.74)	Reference		
Casual	37(48.68)	42(55.26)	1.38	0.72-2.65	0.321
**Comorbidity(ies)**					
No	71(93.42)	69(90.79)	Reference		
Yes	5(6.58)	7(9.21)	1.48	0.44-4.91	0.517
**Mental disorder(s)**					
No	64(84.21)	61(80.26)	Reference		
Yes	12(15.79)	15(19.74)	1.24	0.53-2.91	0.612

*OR = Odds ratio; ^†^IC95% = 95% confidence interval; ^‡^p-value referring to Wald’s chi-square test; ^§^Mean (standard deviation); ^||^Ignored excluded from bivariate analysis

Regarding the clinical, immunological, and laboratory characteristics of adolescents and young adults living with HIV, it was noted that these were new cases, with the average time to initiation of ART after diagnosis among cases and controls being 164.30 days and 117.16 days, respectively. The variables associated with retreatment were diagnosis at the TCC (p=0.255), type of entry into the service (p=0.167), initial ART regimen (p=0.203), side effects of ART (p=0.209), HIV staging according to the WHO (p=0.198), opportunistic infections (p=0.065), and missed appointments before loss to follow-up (p=0.034). The odds ratio for return to treatment was higher among transferred cases (OR: 1.67; 95% CI: 0.80-3.46) and among stage II HIV cases according to the WHO (OR: 1.66; 95% CI: 0.76-3.61) compared to the other categories (Table 2).

**Table 2:** Descriptive and bivariate analysis of clinical, immunological, and laboratory characteristics of adolescents and young adults living with HIV, according to the outcome of retreatment or not after loss to follow-up. Maringá, PR, Brazil, 2024

Variable	Case	Control	Bivariate analysis (crude)		
	n(%)	n(%)	OR*	CI95%^†^	p-value^‡^
**Diagnosis at the TCC^§^**					
No	36(47.37)	31(40.79)		Reference	
Yes	40(52.63)	45(59.21)	0.68	0.35-1.31	0.255
**Admission type**					
New case	58(76.32)	51(67.11)	Reference		
Transferred case	18(23.68)	25(32.89)	1.67	0.80-3.46	**0.167**
**Time to start ART^ǁ^ after diagnosis**				
Continuous variable (in days)	164.30(352.50)^⁋^	117.16(232.74)^⁋^	0.99	0.99-1.00	0.357
**Initial ART^ǁ^ regimen**					
3TC+TDF+DTG	57(75.00)	63(82.89)	Reference		
3TC+TDF+EFZ	14(18.42)	8(10.53)	0.54	0.21-1.39	**0.203**
Other	5(6.58)	5(6.58)	1.18	0.30-4.64	0.805
**Side effects of ART^ǁ^**					
No	60(78.95)	66(86.84)	Reference		
Yes	16(21.05)	10(13.16)	0.56	0.22-1.38	**0.209**
**Number of schemes carried out**					
1	62(81.58)	67(88.16)	Reference		
More than 1	14(18.42)	9(11.84)	0.58	0.22-1.51	0.270
HIV** staging according to the WHO^††/ǁǁ^					
Stage I	41(53.95)	38(50.00)	Reference		
Stage II	16(21.05)	24(31.58)	1.66	0.76-3.61	**0.198**
Stage III	11(14.47)	7(9.21)	0.70	0.24-2.00	0.513
Stage IV	7(9.21)	5(6.58)	0.79	0.23-2.71	0.710
Ignored	1(1.32)	2(2.63)			
**First VL result^‡‡/ǁǁ^**					
Suppressed	59(77.63)	55(72.37)	Reference		
Not suppressed	9(11.84)	10(13.16)	1.21	0.45-3.21	0.696
Undetectable	8(10.53)	9(11.84)	1.40	0.48-4.30	0.528
Ignored	0(0.00)	2(2.63)			
**First CD4+ count^§§^**					
Continuous variable (in cells/mm3)	589.63(304.86)^⁋^	600.81(285.01)^⁋^	1.00	0.99-1.00	0.545
**VL^‡‡^ result before loss to follow-up^ǁǁ^**					
Suppressed	21(27.63)	18(23.68)	Reference		
Not suppressed	10(13.16)	7(9.21)	0.81	0.25-2.58	0.817
Undetectable	45(59.21)	49(64.47)	1.27	0.60-2.69	0.529
Ignored	0(0.00)	2(2.63)			
**CD4+^§§^ count before loss to follow-up**					
Continuous variable (in cells/mm3)	701.47(340.39)^⁋^	656.73(274.99)^⁋^	1.00	0.99-1.00	0.449
**Opportunistic infection(s)^ǁǁ^**					
No	12(15.79)	23(30.26)	Reference		
Yes	64(84.21)	52(68.42)	0.47	0.21-1.04	**0.065**
Ignored	0(0.00)	1(1.32)			
**Sexually transmitted infection^ǁǁ^**					
No	47(61.84)	47(61.84)	Reference		
Yes	29(38.16)	28(36.84)	0.93	0.47-1.81	0.833
Ignored	0(0.00)	1(1.32)			
**Missed appointment(s) before loss to follow-up**					
No	14(18.42)	26(34.21)	Reference		
Yes	62(81.58)	50(65.79)	0.44	0.20-0.93	**0.034**

*OR = Odds ratio; ^*†*^ CI95% = 95% confidence interval; ^‡^p-value referring to Wald’s chi-square test; ^§^TCC = Treatment and Counseling Center; ^ǁ^ART = Antiretroviral therapy; ^⁋^Mean (standard deviation); **HIV = Human Immunodeficiency Virus; ^††^WHO = World Health Organization; ^‡‡^VL = Viral load; ^§§^CD4+ = Immune system cells (lymphocytes); ^ǁǁ^Ignored excluded from bivariate analysis

The final logistic regression model showed a p-value <0.05, indicating statistical significance, for the case group, consisting of seven variables. The odds ratio for retreatment among adolescents and young people living with HIV is 3.46 times higher in the group that has no religion, when compared to the group that has religion (95% CI: 1.52-7.88; p=0.003). The factors that decreased the chances of retreatment were age of 21.91 years at the beginning of follow-up (ORaj: 0.78; 95% CI: 0.62-0.98; p= 0.039), being female (ORaj: 0.12; 95% CI: 0.03-0.45; p= 0.001), living in an institution (ORaj: 0.72; 95% CI: 0.12-0.43; p= 0.004), alcohol use (ORaj: 0.20; 95% CI: 0.08-0.49; p<0.001), having an HIV diagnosis at the TCC (ORaj: 0.31; 95% CI: 0.13-0.74; p=0.008), and missing appointments before loss to follow-up (ORaj: 0.20; 95% CI: 0.07-0.52; p=0.001) (Table 3).

**Table 3:** Multivariate analysis of sociodemographic, behavioral, clinical, immunological and laboratory characteristics of adolescents and young people living with HIV associated with retreatment after loss to follow-up. Maringá, PR, Brazil, 2024

Variable	Multivariate analysis (adjusted)		
	OR*	CI 95%^†^	p-value^‡^
**Age at start of follow-up (in years)**	0.78	0.62-0.98	**0.039**
**Gender**			
Male	Reference		
Female	0.12	0.03-0.45	**0.001**
**Religion**			
No	3.46	1.52-7.88	**0.003**
Yes	Reference		
**Housing**			
With family members	Reference		
With friends	0.70	0.28-1.77	0.464
In an institution	0.72	0.12-0.43	**0.004**
Alone	0.46	0.04-5.47	0.545
**Alcohol use**			
No	Reference		
Yes	0.20	0.08-0.49	**<0.001**
**Diagnosis at the TCC^§^**			
No	Reference		
Yes	0.31	0.13-0.74	**0.008**
**Missed appointment(s) before loss to follow-up**			
No	Reference		
Yes	0.20	0.07-0.52	**0.001**

*OR = Odds ratio; ^†^IC95% = 95% confidence interval; ^‡^p-value referring to the Wald chi-square test; ^§^TCC = Treatment and Counseling Center

For this study, the AUC was 0.82 (95% CI: 0.75-0.88), indicating that the model performs well. In addition, the final predictive model showed high sensitivity (81.6%) and specificity (70.7%).

## Discussion

Among the factors associated with retreatment among adolescents and young people living with HIV, it was evident that the variable of not having a religion was statistically significantly associated with returning to HIV care. The variables of age at the start of follow-up, female gender, alcohol use, living in an institution, HIV diagnosis at the TCC, and missed appointments before abandonment are statistically associated with a lower probability of returning to treatment.

The main results of this study point to the importance of identifying factors associated with re-treatment after loss to follow-up, both those intrinsically related to the life context of each adolescent and young person and those related to treatment. There are still a few international studies addressing this topic at present, given the knowledge gap regarding research focused on returning to HIV care and its relevance to priority populations, such as adolescents and young adults^8^ ^-^ ^9^ ^,^ ^15^.

Retreatment after loss to follow-up is a critical step in the HIV treatment cascade, and it is necessary to search for patients after any treatment failure to facilitate their return^9^. In addition, demographic changes and the predicted increase in the number of young people living with HIV over the next 20 years underscore the urgency of developing scalable models for the provision of HIV prevention services alongside treatment^3^.

It was noted that not having a religion increased the chances of returning to treatment; however, this interpretation should be made with caution, as religion is an organized and/or shared practice or belief. Spirituality, on the other hand, refers to the way individuals relate to the transcendent^16^. The relationship between religion, stigma, and HIV is ambiguous, as evidenced in different cultural contexts^17^ ^-^ ^18^. 

A study conducted in Indonesia identified that conservative religious discourse often reinforces stigma by associating HIV with behaviors considered morally reprehensible, such as homosexuality, drug use, and multiple sexual partners, generating shame, guilt, and stigma^17^. In addition, religious affiliation influences the intensity of stigma, with individuals from conservative traditions showing a greater propensity to stigmatize people living with HIV compared to other religions^18^.

It should be noted that there is still an association between HIV and divine punishment, consequently intensifying discriminatory attitudes and compromising treatment adherence. Thus, religion is a double-edged sword-it can reinforce prejudice when guided by moralistic concepts or contribute to reducing stigma and strengthening care when grounded in discourses of inclusion and solidarity^17^ ^-^ ^18^.

This context may also be explained by the observation that people who do not have a religion trust medicine more and thus maintain treatment or return to it. However, the desire to place life under the principles of religiosity may have originated in the belief that it could lead to recovery from a complex health condition, such as HIV^16^.

A study conducted in Zimbabwe with young people living with HIV showed that placing one’s life under God’s guidance in relation to religion was significantly linked to an increase in treatment failure^16^.

In addition, another explanation is the fact that living with HIV is often associated with sinful behavior, and young people living with HIV, because they were not considered innocent, were also not worthy of protection (by church authorities)^16^, causing them to abandon religion and seek treatment.

Among the factors analyzed for their negative influence on retreatment, age at the start of follow-up was found to reduce the likelihood of return. However, further investigation of additional potential determinants is necessary, since psychosocial support, family support, and educational interventions are important during youth, providing a more holistic understanding of treatment adherence behaviors^19^.

In addition, adolescents and young people living with HIV are influenced by external conditions closely associated with them, such as forgetfulness and mental health problems, contributing to suboptimal adherence^20^.

Findings indicated that being female reduced the chances of adolescent and young patients living with HIV returning to treatment. The gender difference may be related to the fact that women access HIV testing services more than men, especially during prenatal care^11^ ^,^ ^21^ *.* It is noteworthy that women who become pregnant adhere to treatment because they want to protect their babies. A study conducted in southern Brazil found that being pregnant increased the chances of adherence to HIV treatment^22^ ^-^ ^23^.

As for women not returning to treatment, this may be due to the challenges of being the primary caregivers for children and other family members, especially when they are ill. The fact that they take care of their family members’ health often interferes with their attendance at appointments and their ART routine^11^ ^,^ ^21^ *.*

The fact that women do not have paid work may also lead them to avoid attending appointments and collecting their ART^21^. In addition, many women find it difficult to disclose their HIV status and therefore need to hide while taking ART, which means they end up forgetting to take their medication^22^.

It should be noted that gender inequalities have a direct impact on women’s access to and treatment adherence. In addition, they face the burden of social roles, difficulty in negotiating sexual relations, economic dependence, and the moral stigma associated with female HIV status, resulting in problems not only in initial adherence but also in retreatment. Therefore, the feminization of HIV highlights not only the increase in cases among women, but also the symbolic and structural barriers that compromise their continuity in care^24^ ^-^ ^25^.

Living in a drug treatment facility and/or detention center also creates a barrier to HIV treatment due to the lack of personal housing. Therefore, it is necessary to increase engagement in care among people living with HIV, reviewing service delivery models and strengthening the reach of care by addressing housing, harm reduction, and treatment of substance use disorders, as well as specific sex and gender interventions^25^.

A study conducted in Canada shows that homelessness was associated with a 44% reduction in the chances of overall progression through the HIV care cascade, with a 41% reduction in the chances of receiving ART and a 54% reduction in maintaining adherence and achieving viral load suppression. Given this, there is a reinforced need for service integration to address the intersectional challenges of HIV among the most vulnerable populations^26^.

In addition, it is essential to encourage the creation of a consolidated support network for PLHIV so that they can become more persistent, more confident, and better adhere to treatment^27^.

Building social support among family and friends, especially when community prejudice and stigma are substantial, is a powerful strategy for social normalization^28^ ^-^ ^29^. Therefore, healthcare professionals should encourage their patients to create new social relationships both with those who accept the HIV diagnosis and with other PLHIV^28^. Thus, during the nursing consultation in the specialized service, the nurse should identify and map the patient’s current support network to develop a care plan that includes them^27^.

Alcohol use was also a factor that hindered the return to HIV care, due to impaired prospective memory and interactive behavior of toxicity/avoidance beliefs due to the deterioration of physical health and social behavior. As a result, alcohol dependence develops, whose abstinence causes adverse effects and thus can lead to intentional and unintentional non-adherence^30^ ^-^ ^31^.

Another situation that can interfere with retreatment is the stigmatization of alcohol use among PLHIV who are on ART, that is, society perceives alcohol use as shameful and irresponsible due to the individual’s behavior, thus aggravating the adverse effects of alcohol use on treatment adherence, compromising treatment efficacy, and disease progression^32^ ^-^ ^33^.

Therefore, health services must be attentive to alcohol abuse among PLHIV, especially adolescents and young people, who, given the characteristics of their age, already engage in risky health behaviors. That said, care must be integrated and able to screen, treat, and monitor alcohol abuse and HIV, thereby reducing the treatment gap and the poor outcomes of alcohol use at all stages of HIV care^32^ ^-^ ^33^.

The diagnosis at the TCC also influences the return of HIV care, and even though the service where this study was conducted offers counseling, adolescents and young people themselves must deal with the stigma that compromises their ability to take ART openly and without fear. And, with compromised adherence, unsuppressed viral load is almost inevitable. Therefore, counseling should emphasize the chronic nature of the disease and the need for lifelong ART treatment^34^.

A study conducted in Uganda shows that good adherence to ART is crucial for viral suppression, yet many young people are unaware of this**.** In addition, adolescents who never achieved viral suppression after starting treatment expressed concerns about not knowing the rationale for taking pills regularly^34^, so this context may also influence retreatment.

Missing appointments before dropping out is a factor that reduces the chances of retreatment among adolescents and young people living with HIV, and patients who exhibit this behavior are considered non-compliant with treatment by health professionals and the health system in general^21^.

Adolescents and young people living with HIV require lifelong treatment for HIV, making medical appointments, regular blood tests, and unpleasant medications crucial^35^. In addition, they address physical, emotional, and social contexts that affect decision-making and behaviors related to follow-up and treatment adherence. Furthermore, loss to follow-up also worsens from early adolescence to late youth/early adulthood, possibly due to reduced caregiver involvement and increased responsibility and autonomy^35^.

One strategy to reduce missed appointments and ART withdrawals is to prescribe several months’ worth of medication (3 or 6 months) or use new technologies, such as long-acting injectable ART, which can make it easier for young people to balance the priorities of HIV care and life outside specialized services^21^.

In addition, strategies to improve patient resilience and outreach after any treatment gap can facilitate return and increase awareness among managers and health professionals of patients’ efforts to remain in follow-up^21^ ^,^ ^36^.

Regarding the limitations of the study, only secondary data from adolescents’ and young people’s medical records were used, which may lead to incomplete information and, consequently, interpretation bias.

The implications for advancing scientific knowledge in health and nursing include developing a study that addresses a knowledge gap in this population. The results allow nurses and other health professionals to create a care plan tailored to the needs of adolescent and young patients, to prevent permanent abandonment of treatment. Future studies on re-engagement, in other words, the return to treatment of adolescents and young adults living with HIV, are suggested, focusing on these predictors to investigate their causal mechanisms in re-treatment.

## Conclusion 

The return to treatment of adolescents and young people living with HIV has multifactorial determinants associated with sociodemographic, behavioral, and clinical profiles. It was noted that not practicing religion interferes with withdrawal, and the factors negatively associated with this group’s return to service were being female, using alcohol, living in an institution, being diagnosed at the TCC, and missing appointments before losing follow-up. These results provide insights into the need to review health practices to prevent permanent abandonment and promote the retention of adolescents and young people in HIV treatment. In addition, there is a need for practical actions, such as strengthening external public policies aimed at housing, greater inclusion of women (not only pregnant women), and reducing prejudice in HIV prevention, treatment, and care strategies among adolescents and young people.

## Data Availability

All data generated or analysed during this study are included in this published article.

## References

[1] United Nations Children’s Fund (2024). Global and regional trends: although strides have been made in the HIV response, children are still affected by the epidemic.

[2] Rungmaitree S, Thamniamdee N, Sachdev S, Phongsamart W, Lapphra K, Wittawatmongkol O (2022). The outcomes of transition from pediatrics to adult care among adolescents and young adults with HIV at a tertiary care center in Bangkok. J Int Assoc Provid AIDS Care.

[3] Shahmanesh M, Chimbindi N, Busang J, Chidumwa G, Mthiyani N, Herbst C (2024). Effectiveness of integrating HIV prevention within sexual reproductive health services with or without peer support among adolescents and young adults in rural KwaZulu-Natal, South Africa (Isisekelo Sempilo): 2×2 factorial, open-label, randomised controlled trial. Lancet HIV.

[4] Smith T, Seeley J, Shahmanesh M, Psaros C, Munikwa C, Ngwenya N (2023). Influences on decision-making about disclosure of HIV status by adolescents and young adults living with HIV in KwaZulu-Natal, South Africa. Afr J AIDS Res.

[5] Toska E, Zhou S, Chen-Charles J, Gittings L, Operario D, Cluver L (2023). Factors associated with preferences for long-acting injectable antiretroviral therapy among adolescents and young people living with HIV in South Africa. AIDS Behav.

[6] Haas AD, Lienhard R, Didden C, Cornell M, Folb N, Boshomane TMG (2023). Mental health, ART adherence, and viral suppression among adolescents and adults living with HIV in South Africa: a cohort study. AIDS Behav.

[7] Piran CMG, Magalhães LG, Shibukawa BMC, Rissi GP, Merino MDFGL, Furtado MD (2023). Treatment non-adherence or abandonment among adolescents and young individuals living with HIV/AIDS: a scoping review. Aquichan.

[8] Beres LK, Mwamba C, Bolton-Moore C, Kennedy CE, Simbeza S, Topp SM (2023). Trajectories of re-engagement: factors and mechanisms enabling patient return to HIV care in Zambia. J Int AIDS Soc.

[9] Martinez-Guerra BA, Valdez-Ventura R, Caro-Vega Y, Sierra-Madero JG, Crabtree-Ramírez BE (2023). Gaps in the continuum of care in HIV-positive adults and the need for caution in those returning to care after loss to follow-up. AIDS Care.

[10] Mtisi EL, Mushy SE, Mkawe SG, Ndjovu A, Mboggo E, Mlay BS (2023). Risk factors for interruption in treatment among HIV-infected adolescents attending health care and treatment clinics in Tanzania. AIDS Res Ther.

[11] Tesha ED, Kishimba R, Njau P, Revocutus B, Mmbaga E (2022). Predictors of loss to follow-up from antiretroviral therapy among adolescents with HIV/AIDS in Tanzania. PLoS One.

[12] von Elm E, Altman DG, Egger M, Pocock SJ, Gøtzsche PC, Vandenbroucke JP (2007). The Strengthening the Reporting of Observational Studies in Epidemiology (STROBE) statement: guidelines for reporting observational studies. BMJ.

[13] World Health Organization (2017). Adolescent health.

[14] Biblioteca Virtual de Saúde, Descritores em CIências da Saúde (2024). Retratamento.

[15] Calabrese S, Perkins M, Lee S, Allison S, Brown G, Jean-Philippe P (2023). Adolescent and young adult research across the HIV prevention and care continua: an international programme analysis and targeted review. J Int AIDS Soc.

[16] Wüthrich-Grossenbacher U (2024). Young people living with HIV in Zimbabwe use the conventional, religious, and traditional health systems in parallel: findings from a mixed methods study. Religions.

[17] Hutahaean BSH, Stutterheim SE, Jonas KJ (2025). Religion, faith, and spirituality as barriers and facilitators to antiretroviral therapy initiation among people with HIV in Indonesia. AIDS Patient Care STDS.

[18] Lane BL, Sabuncu C, Yang Y, Okantey B, Campbell DN, Bryant TR (2025). Discrimination and mental health among Black and Latino people living with HIV: understanding the role of religion and spirituality. AIDS Behav.

[19] Mtisi TJ, Kouamou V, Morse GD, Dzinamarira T, Ndhlovu CE (2024). Comparing pill counts and patient self-reports versus DBS tenofovir concentrations as ART adherence measurements with virologic outcomes and HIV drug resistance in a cohort of adolescents and young adults failing ART in Harare, Zimbabwe. J Infect Public Health.

[20] Nantambi LB, Suubi M (2023). Assessment of factors affecting adherence to antiretroviral therapy among HIV-infected adolescents attending ART clinic at Kajjansi Health Centre IV, Wakiso District: a cross-sectional study. Stud J Health Res Afr..

[21] Chamberlin S, Mphande M, Phiri K, Kalande P, Dovel K (2022). How HIV clients find their way back to the ART clinic: a qualitative study of disengagement and re-engagement with HIV care in Malawi. AIDS Behav.

[22] Zurbachew Y, Hiko D, Bacha G, Merga H (2023). Adolescents’ and youths’ adherence to antiretroviral therapy for better treatment outcome and its determinants: multi-center study in public health facilities. AIDS Res Ther.

[23] Martins RS, Knauth DR, Vigo A, Fisch P (2023). Marker events associated with adherence to HIV/AIDS treatment in a cohort study. Rev Saude Publica.

[24] Lourenço GO, Amazonas MCLA, Lima RDM (2018). Neither a saint nor a whore, just a woman: the feminization of HIV/AIDS and the seropositivity experience. Sex Salud Soc.

[25] Sharp A, Sorokopud-Jones M, Haworth-Brockman M, Kasper K, MacKenzie L, Ireland L (2024). Sex differences in houselessness, injection drug use, and mental health conditions among people newly diagnosed with HIV in Manitoba, Canada from 2018 to 2021: a retrospective cohort study. Lancet Reg Health Am.

[26] Reddon H, Fairbairn N, Grant C, Milloy MJ (2023). Experiencing homelessness and progression through the HIV cascade of care among people who use drugs. AIDS.

[27] andu JBS, Teston EF, Andrade GKS, Marcon SS. (2022). Coping with the health condition from the perspective of people with HIV who abandoned treatment. Rev Bras Enferm.

[28] Roy M, Czaicki N, Holmes C, Chavan S, Tsitsi A, Odeny T (2016). Understanding sustained retention in HIV/AIDS care and treatment: a synthetic review. Curr HIV/AIDS Rep..

[29] Souza VB, Lima ALS, Harmuch C, Bertozzi LC, Ignachewski AJ, Barbosa C (2024). Social representations of primary care nurses on “being young” and prevention of HIV. Cienc Cuid Saude.

[30] Woolf-King SE, Sheinfil AZ, Ramos J, Foley JD, Moskal D, Firkey M, A use, review adherence, action theoretical (2022). Health Psychol Rev.

[31] Lopez CM, Moreland A, Goodrum NM, Davies F, Meissner EG, Danielson CK (2023). Association of mental health symptoms on HIV care outcomes and retention in treatment. Gen Hosp Psychiatry.

[32] Perazzo H, Gonçalves JL, Cardoso SW, Grinsztejn B, Veloso VG, Luz PM (2024). Pathways to poor adherence to antiretroviral therapy among people living with HIV: the role of food insecurity and alcohol misuse. AIDS Behav.

[33] Aurpibul L, Kosalaraksa P, Kawichai S, Lumbiganon P, Ounchanum P, Songtaweesin WN (2023). Alcohol use, suicidality and virologic non-suppression among young adults with perinatally acquired HIV in Thailand: a cross-sectional study. J Int AIDS Soc.

[34] Izudi J, Cattamanchi A, Castelnuovo B, King R (2024). Barriers and facilitators to viral load suppression among people living with HIV following intensive adherence counseling in Kampala, Uganda: a qualitative study. Soc Sci Med.

[35] Yusuf H, Agwu A (2021). Adolescents and young adults with early acquired HIV infection in the United States: unique challenges in treatment and secondary prevention. Expert Rev Anti Infect Ther.

[36] Beres LK, Schwartz S, Simbeza S, McGready J, Eshun-Wilson I, Mwamba C (2021). Patterns and predictors of incident return to HIV care among traced, disengaged patients in Zambia: analysis of a prospective cohort. J Acquir Immune Defic Syndr.

