# Supplementary material for: Retreatment after loss of follow-up in adolescent and young adult patients living with HIV: a case-control study
Source: Rev Lat Am Enfermagem. 2026 Jun 15;34:e4801. doi: 10.1590/1518-8345.7973.4801 (PMC13277353; doi:10.1590/1518-8345.7973.4801)
Supplement: Translation

# Translation: Retratamento após perda de seguimento em pacientes adolescentes e jovens vivendo com HIV: estudo caso-controle^*^

**DOI:** 10.1590/1518-8345.7973.4802

**Authors:** Camila Moraes Garollo Piran, Alana Vitória Escritori Cargnin, Mariana Martire Mori, Rosana Rosseto de Oliveira, João Manuel Graça Frade, Marcela Demitto Furtado

**Affiliations:** 1 Universidade Estadual de Maringá, Maringá, PR, Brasil.; 2 Polytechnic University of Leiria, Leiria, Portugal.; 3 Scholarship holder at the Coordenação de Aperfeiçoamento de Pessoal de Nível Superior (CAPES), Brasil.

**Keywords:** Retratamento, Perda de Seguimento, Adolescentes, Adulto Jovem, HIV, Síndrome da Imunodeficiência Adquirida.

## Abstract

**Objetivo:**

identificar os fatores associados ao retratamento após perda de seguimento entre adolescentes e jovens vivendo com Vírus da Imunodeficiência Humana.

**Método:**

estudo de caso-controle pareado (proporção 1/1) com coleta de dados em prontuários. Os casos foram de adolescentes e jovens, com diagnóstico referente ao Vírus da Imunodeficiência Humana/Síndrome da Imunodeficiência Adquirida com retratamento após perda de seguimento. Os controles foram adolescentes e jovens diagnosticados, em perda de seguimento, sem histórico de retratamento. Para análise estatística, adotou-se regressão logística.

**Resultados:**

76 casos e 76 controles foram incluídos. A variável associada à maior chance de retratamento foi não ter religião (ORaj:3,46; IC95%:1,52-7,88; p=0,003). Já os fatores que diminuíram as chances de retratamento foram 21,91 anos de idade no início do acompanhamento (ORaj:0,78; IC95%:0,62-0,98; p=0,039), sexo feminino (ORaj:0,12; IC95%:0,03-0,45; p=0,001), morar em instituição (ORaj:0,72; IC95%:0,12-0,43; p=0,004), uso de álcool (ORaj:0,20; IC95%:0,08-0,49; p<0,001), diagnóstico de Vírus da Imunodeficiência Humana no Centro de Tratamento e Aconselhamento (ORaj:0,31; IC95%:0,13-0,74; p=0,008) e falta(s) em consulta antes da perda de seguimento (ORaj:0,20; IC95%:0,07-0,52; p=0,001).

**Conclusão:**

o retorno ao tratamento de adolescentes e jovens vivendo com Vírus da Imunodeficiência Humana possui determinantes multifatoriais associados ao perfil sociodemográfico, comportamental e clínico.

## Destaques

**(1)** Primeiro estudo brasileiro abordando retratamento entre jovens vivendo com HIV. **(2)** A ausência de prática religiosa esteve associada a maior chance de retratamento. **(3)** Sexo, uso de álcool e morar em instituição implicam no retratamento. **(4)** Diagnóstico no CTA e falta(s) em consulta diminuíram as chances de retratamento. **(5)** Fornece *insights* sobre a prevenção do abandono permanente.

## Introdução

Estima-se que, em 2023, havia 40 milhões de pessoas vivendo com o Vírus da Imunodeficiência Humana (HIV) (PVHIV), sendo que a cada sete novas infecções por HIV duas são entre adolescentes e jovens^1^. Mesmo com a terapia antirretroviral (TARV) desempenhando impacto relevante na redução da morbimortalidade entre PVHIV, os adolescentes e jovens são mais expostos a contextos negativos^2^, uma vez que este grupo etário muitas vezes está ausente na cascata de tratamento do HIV^3^.

A inclusão da TARV fez com que o HIV se tornasse uma condição crônica de saúde controlável, proporcionando melhor qualidade de vida, retorno à expectativa de vida normal e impactos substanciais na prevenção e tratamento do HIV. No entanto, para os adolescentes e jovens que vivem com essa condição, ainda é desafiador manter a adesão ao tratamento em decorrência do uso diário da TARV ao longo da vida^4^ ^-^ ^6^.

A não adesão contínua ou perda de seguimento provoca longas interrupções do tratamento ou descontinuação da TARV, sendo uma das principais barreiras para acabar com a aids, pois pode levar à resistência aos medicamentos e, consequentemente, insucesso do tratamento^5^ ^-^ ^6^. Além disso, adolescentes e jovens vivendo com HIV têm resultados piores em relação ao tratamento de HIV quando comparados a outras faixas etárias que vivem com HIV, devido aos comportamentos de risco à saúde que apresentam nesta faixa etária^5^.

Posto isto, percebe-se que muitos adolescentes e jovens vivendo com HIV abandonam o tratamento^7^ ^)^ e, após um determinado período, retornam ao serviço^8^. O retorno dos cuidados com HIV após perda de seguimento tem se tornado um comportamento de saúde comum, porém ainda pouco compreendido. O retratamento é cada vez mais presente na cascata de cuidados de HIV, nos quais muitos indivíduos entram e saem dos cuidados após início de tratamento e ao longo da vida^8^ ^-^ ^9^.

Embora os fatores que impulsionam a perda de seguimento ou abandono sejam estudados^7^ ^,^ ^10^ ^-^ ^11^, pouco se sabe sobre os fatores que facilitam e prejudicam o retorno ao tratamento^8^ ^-^ ^9^. Os resultados desse público em específico ainda são incipientes e, como consequência, impossibilitam os profissionais de saúde, como os enfermeiros que estão no gerenciamento dos serviços, de identificar os fatores associados ao retratamento e de implementar estratégias preventivas para evitar um abandono permanente^8^.

Explorar o retratamento após perda de seguimento em um local que realiza o tratamento em diferentes contextos poderá contribuir para a implementação de novas estratégias para melhorar os resultados entre adolescentes e jovens. Isso será crucial para o cumprimento das metas globais de eliminação da aids até 2030. Assim, este estudo pretende responder à seguinte questão de pesquisa: quais os fatores associados ao retorno dos cuidados com HIV entre adolescentes e jovens? Posto isto, o objetivo deste estudo foi identificar os fatores associados ao retratamento após perda de seguimento entre adolescentes e jovens vivendo com HIV.

## Método

### Tipo de estudo

Estudo epidemiológico, do tipo caso-controle pareado com proporção 1 caso/1 controle de adolescentes e jovens vivendo com HIV, o qual estava aninhado a uma coorte ambispectiva. A proporção de 1:1 foi estabelecida diante do tempo hábil para execução desta pesquisa. O *Strengthening the Reporting of Observational Studies in Epidemiology* (STROBE) foi utilizado para reportar o estudo como recomendado pela *Enhancing the Quality and Transparency of Health Research* (EQUATOR *Network*)^12^.

### Local do estudo

O local do estudo foi o Serviço de Atenção Especializada (SAE) pertencente ao Ambulatório de Infecções Sexualmente Transmissíveis/HIV/aids da 15ª Regional de Saúde, localizado no noroeste do Estado do Paraná, Brasil. Trata-se de um serviço público, especializado e de referência que presta atendimento a Maringá e mais 29 municípios. 

### População

Foram definidos como casos os adolescentes e jovens com diagnóstico referente ao HIV/aids, segundo a Classificação Internacional de Doenças (CID-10) - indicador de estatísticas dos agravos de mortalidade e morbidade - representado pelos códigos B20.0 a B24, com posterior retratamento após perda de seguimento. Os controles foram adolescentes e jovens com CID-10 referente ao HIV/aids, em perda de seguimento no SAE após iniciar a TARV, sem histórico de retratamento. 

Foram considerados os seguintes critérios de inclusão: ter idade entre 10 e 24 anos, ter adquirido o HIV por transmissão sexual e ter iniciado a TARV. Destaca-se que as faixas etárias de 10 a 19 anos e 20 a 24 anos correspondem aos períodos de adolescência e juventude, respectivamente^13^. Definiram-se como critérios de não inclusão: pacientes que não iniciaram a TARV e os que adquiriram HIV por transmissão vertical. E os critérios de exclusão foram: pacientes que evoluíram a óbito e quando não existia caso ou controle para o pareamento.

### Definição da amostra

Foi utilizada a amostra total de 198 adolescentes e jovens vivendo com HIV que estavam em situação de perda de seguimento, atendidos no serviço entre o período de janeiro de 2017 e dezembro de 2023. Após a análise exploratória dos prontuários dos pacientes adolescentes e jovens, averiguando os critérios de seleção supracitados, foi realizado o emparelhamento amostral, buscando a maior similaridade possível entre os indivíduos. 

Os grupos foram pareados pela faixa etária e ano de entrada no serviço. A variável faixa etária foi estabelecida no pareamento para proporcionar maior similaridade entre as idades dos casos e controles; mesmo que algumas idades não fossem exatamente as mesmas entre alguns pares, ainda eram próximas. Não foram incluídos 19 pacientes que não iniciaram a TARV e cinco pacientes que adquiriram HIV por transmissão vertical. Foram excluídos três óbitos e 19 pacientes que eram casos, devido à falta de controles dentro do período do estudo (Figura 1).

**Figura 1:** Seleção dos prontuários dos adolescentes e jovens vivendo com HIV. Maringá, PR, Brasil, 2024

### Variáveis do estudo 

As informações dos pacientes referentes a cada variável foram coletadas nos prontuários de saúde. A variável dependente (retratamento após perda de seguimento) foi adotada para a regressão logística, com a finalidade de verificar a associação com as variáveis independentes.

*Variável dependente*

Adotou-se como variável dependente (desfecho) o retratamento após perda de seguimento. O retratamento ou retorno aos cuidados é definido como tratamento do paciente após o abandono, com a repetição do mesmo tratamento inicial, ou com uma medida adicional ou alternativa. O retratamento muitas vezes é utilizado com referência a uma modalidade diferente, quando a original foi usada de forma inadequada, prejudicial ou não apresentou sucesso^8^ ^-^ ^9^ ^,^ ^14^.

*Variáveis independentes*

Como variáveis independentes foram agrupadas as características:

- Sociodemográficas: idade no momento do diagnóstico (em anos), idade no início do acompanhamento (em anos), sexo (masculino; feminino), estado civil [sem companheiro(a), com companheiro(a)], raça/cor (branca; não branca), escolaridade (<12 anos; ≥12 anos), religião (não; sim), orientação sexual (heterossexual; homossexual/bissexual), ocupação (empregado/autônomo; estudante/do lar; desempregado), moradia (com familiares; com amigos(as); em instituição; sozinho(a); ignorada);
- Comportamentais: uso de álcool (não; sim), uso de tabaco (não; sim), uso de drogas (não; sim), parceria sexual (fixa; casual), comorbidade(s) (não; sim), transtorno(s) mental(is) (não; sim);
- Clínicas, imunológicas e laboratoriais: diagnóstico no Centro de Tratamento e Aconselhamento (CTA) (não; sim), tipo de entrada (caso novo; caso transferido), tempo de início da TARV após diagnóstico (em dias), regime inicial da TARV [Lamivudina (3TC) + Tenofovir (TDF) + Dolutegravir (DTG); Lamivudina (3TC) + Tenofovir (TDF) + Efavirenz (EFZ); Outro], efeito colateral da TARV (não; sim), número de esquemas realizados (1; mais que 1) estadiamento do HIV segundo a OMS (estágio I; estágio II; estágio III; estágio IV; ignorado), primeiro resultado de carga viral (CV) (suprimida; não suprimida; indetectável; ignorado), primeira contagem de linfócitos T auxiliares CD4+ (CD4+) (em cel/mm^3^), resultado de CV antes da perda de seguimento (suprimida; não suprimida; indetectável; ignorado) contagem de CD4+ antes da perda de seguimento (em cel/mm^3^), infecção(ões) oportunista(s) (não; sim; ignorado), Outra infecção sexualmente transmissível(não; sim; ignorado), falta(s) em consulta antes da perda de seguimento (não; sim);

### Instrumentos utilizados para a coleta das informações

Os dados foram coletados de fontes secundárias utilizando os prontuários de saúde disponibilizados no ambulatório de Infecções Sexualmente Transmissíveis/HIV/aids. O instrumento de coleta de dados foi estabelecido conforme recomendado pelo Ministério da Saúde, estando ele de acordo com a ficha de notificação e acompanhamento ambulatorial de casos de HIV. Para garantir a comparabilidade dos dados, foi utilizado o mesmo instrumento para os casos e os controles.

### Coleta de dados

Os dados foram coletados entre junho e outubro de 2024, contando com duas assistentes de pesquisa, sendo uma mestranda e outra doutoranda, ambas participantes do projeto de pesquisa. As assistentes de pesquisa foram treinadas*in loco*pela pesquisadora principal e auxiliaram na coleta e dupla checagem dos dados com a finalidade de garantir a veracidade das informações. Ambas foram cegadas para os objetivos da pesquisa tanto quanto para a questão do estudo, como estratégia para minimizar viés de confusão e evitar relações indevidas entre as respostas. As informações foram inseridas e armazenadas em um banco de dados gerenciado pela pesquisadora principal. Além do pareamento, a variável de registro e a variável do número do prontuário foram utilizadas como informação adicional para melhor diferenciar os prontuários e minimizar o viés de seleção.

### Tratamento e análise dos dados 

Para tratamento dos dados, foi utilizado o *Microsoft Excel* ^*®*^ e depois os dados foram exportados para o *software SPSS Statistics* ^*®*^ , versão 25.0, para realização da análise dos dados. A princípio, foram descritas as características sociodemográficas, comportamentais, clínicas, imunológicas e laboratoriais de adolescentes e jovens vivendo com HIV, segundo o desfecho de retratamento ou não após perda de seguimento, utilizando-se medidas de frequência absoluta e relativa, tendência central (média) e dispersão (desvio-padrão). Em seguida, para verificar os fatores associados ao reingresso no tratamento entre adolescentes e jovens que haviam o descontinuado, empregaram-se modelos de regressão logística binomial.

Realizaram-se análises bivariadas para comparar cada variável com o desfecho em estudo. Aquelas que tiveram p-valor≤0,25 no teste qui-quadrado de Wald foram avaliadas em relação à multicolinearidade, a fim de que modelos multivariados parcimoniosos e robustos fossem construídos. Em seguida, modelos múltiplos foram testados por meio da metodologia de seleção *stepwise backward* de variáveis. Foram preservadas nos modelos finais as variáveis independentes com p-valor<0,05 no referido teste, quando ajustadas entre si.

Como medida de associação, calcularam-se as razões de chance (*odds ratios*, OR) e razões de chance ajustada (ORaj) acompanhadas de seus intervalos de confiança de 95% (IC95%). Essas razões representaram a chance aumentada ou reduzida de cada subvariável para a ocorrência do retratamento após perda de seguimento em relação às subcategorias de referência nas respectivas variáveis independentes. O modelo final foi considerado significativo em comparação ao nulo no teste *omnibus*. 

Para o modelo múltiplo final, foi realizada uma análise de *receiver operating characteristic* (ROC, característica de operação do receptor), uma maneira útil de avaliar a precisão das predições do modelo. A partir disso, calculou-se a *area under the curve* (AUC, área sob a curva), representando a probabilidade de que a predição esteja na ordem correta ao observar uma variável de teste. Além disso, a sensibilidade e a especificidade do modelo final foram calculadas no ponto de corte ideal, definido pelo índice de Youden.

## Aspectos éticos

Mesmo que não tenha ocorrido contato direto com os participantes, os prontuários possuíam informações sigilosas. Assim, atendendo às Resoluções n.º 466/12 e 510/16 do Conselho Nacional de Saúde (CNS), o estudo foi submetido ao Comitê Permanente de Ética em Pesquisa Envolvendo Seres Humanos da instituição signatária, sendo aprovado sob pareceres n.º 5.202.623/2022 e n.º 6.897.755/2024, e Certificado de Apresentação de Apreciação Ética (CAAE): 52331221.3.0000.0104.

## Resultados

Foram analisados 198 prontuários de adolescentes e jovens vivendo com HIV, sendo elegíveis para o estudo 152, correspondendo a 76 casos (retratamento) e 76 controles (não retratamento). Em relação às características sociodemográficas e comportamentais dos adolescentes e jovens dos casos e controles, a idade média no diagnóstico foi de 21,39 anos e 21,65 anos, respectivamente. Em ambos os grupos, a maioria era do sexo masculino e sem companheiro. 

Identificou-se associação estatisticamente significativa entre o retratamento e as variáveis idade no início do acompanhamento (p=0,062), sexo (p=0,010), raça/cor (p=0,233), religião (p=0,003), ocupação (p=0,114), moradia (p=0,032) e uso de álcool (p=0,022). A chance de ocorrência de retratamento após perda de seguimento foi maior entre indivíduos de raça/cor não branca (OR: 1,55; IC95%: 0,75-3,18), os que não têm religião (OR: 2,70; IC95%: 1,39-5,25), que estão desempregados (OR: 3,04; IC95%: 0,76-12,12) e moram em instituição (OR: 0,17; IC95%: 0,03-0,86) (Tabela 1).

**Tabela 1:** Análise descritiva e bivariada das características sociodemográficas e comportamentais de adolescentes e jovens vivendo com HIV, segundo o desfecho de retratamento ou não após perda de seguimento. Maringá, PR, Brasil, 2024

Variável	Caso	Controle	Análise bivariada (bruta)		
	n(%)	n(%)	OR*	IC95%^†^	p-valor^‡^
**Idade no momento do diagnóstico**				
Variável contínua (em anos)	21,39(1,96)^§^	21,65(2,00)^§^	1,05	0,89-1,25	0,413
**Idade no início do acompanhamento**				
Variável contínua (em anos)	21,91(1,84)^§^	22,47(1,79)^§^	0,84	0,98-1,42	**0,062**
**Sexo**					
Masculino	56(73,68)	69(90,79)	Referência		
Feminino	20(26,32)	7(15,79)	0,29	0,11-0,74	**0,010**
**Estado civil**					
Sem companheiro(a)	60(78,95)	65(85,53)	Referência		
Com companheiro(a)	16(21,05)	11(14,47)	0,65	0,28-1,52	0,326
**Raça/cor**					
Branca	58(76,32)	51(67,11)	Referência		
Não branca	18(23,68)	25(32,89)	1,55	0,75-3,18	**0,233**
**Escolaridade**					
<12 anos	26(34,21)	24(31,58)	Referência		
≥12 anos	50(65,79)	52(68,42)	1,08	0,54-2,14	0,818
**Religião**					
Não	29(38,16)	48(63,16)	2,70	1,39-5,25	**0,003**
Sim	47(61,84)	28(36,84)	Referência		
**Orientação sexual**					
Heterossexual	23(30,26)	17(22,37)	Referência		
Homossexual/bissexual	53(69,74)	59(77,63)	1,45	0,70-3,02	0,313
**Ocupação**					
Empregado/autônomo	57(75,00)	50(65,79)	Referência		
Estudante/do lar	16(21,05)	18(23,68)	1,14	0,51-2,52	0,741
Desempregado	3(3,95)	8(10,53)	3,04	0,76-12,12	**0,114**
**Moradia^||^**					
Com familiares	49(64,47)	36(10,53)	Referência		
Com amigos(as)	2(2,63)	12(15,79)	1,42	0,69-2,90	0,337
Em instituição	2(2,63)	3(3,95)	0,17	0,03-0,86	**0,032**
Sozinho(a)	23(30,26)	24(31,58)	0,69	0,01-4,55	0,705
Ignorada	0(0,00)	1(1,32)			
**Uso de álcool**					
Não	25(32,89)	39(51,32)	Referência		
Sim	51(67,11)	37(48,68)	0,46	0,23-0,89	**0,022**
**Uso de tabaco**					
Não	46(60,53)	47(61,84)	Referência		
Sim	30(39,47)	29(38,16)	0,88	0,45-1,70	0,706
**Uso de drogas**					
Não	52(68,42)	54(71,05)	Referência		
Sim	24(31,58)	22(28,95)	0,85	0,42-1,73	0,669
**Parceria sexual**					
Fixa	39(51,32)	34(44,74)	Referência		
Casual	37(48,68)	42(55,26)	1,38	0,72-2,65	0,321
**Comorbidade(s)**					
Não	71(93,42)	69(90,79)	Referência		
Sim	5(6,58)	7(9,21)	1,48	0,44-4,91	0,517
**Transtorno(s) mental(is)**					
Não	64(84,21)	61(80,26)	Referência		
Sim	12(15,79)	15(19,74)	1,24	0,53-2,91	0,612

*OR = *Odds ratio;* ^†^IC95% = Intervalo de confiança de 95%; ^‡^p-valor referente ao teste qui-quadrado de Wald; ^§^Média (desvio-padrão); ^||^Ignorados excluídos da análise bivariada

Acerca das características clínicas, imunológicas e laboratoriais dos adolescentes e jovens vivendo com HIV, notou-se que se tratavam de casos novos, sendo o tempo médio de início da TARV após o diagnóstico entre os casos e controles de 164,30 dias e 117,16 dias, respectivamente. As variáveis associadas ao retratamento foram: diagnóstico no CTA (p= 0,255), tipo de entrada no serviço (p=0,167), regime inicial da TARV (p=0,203), efeito colateral da TARV (p=0,209), estadiamento do HIV segundo a OMS (p=0,198), infecções oportunistas (p=0,065) e falta(s) em consultas antes da perda de seguimento (p=0,034). A razão de chance do retorno ao tratamento foi maior entre o caso transferido (OR: 1,67; IC95%: 0,80-3,46) e estágio II do HIV segundo a OMS (OR: 1,66; IC95%: 0,76-3,61) quando comparados às outras categorias (Tabela 2).

**Tabela 2:** Análise descritiva e bivariada das características clínicas, imunológicas e laboratoriais de adolescentes e jovens vivendo com HIV, segundo o desfecho de retratamento ou não após perda de seguimento. Maringá, PR, Brasil, 2024

Variável	Caso	Controle	Análise bivariada (bruta)		
	n(%)	n(%)	OR*	IC95%^†^	p-valor^‡^
**Diagnóstico no CTA^§^**					
Não	36(47,37)	31(40,79)		Referência	
Sim	40(52,63)	45(59,21)	0,68	0,35-1,31	0,255
**Tipo de entrada**					
Caso novo	58(76,32)	51(67,11)	Referência		
Caso transferido	18(23,68)	25(32,89)	1,67	0,80-3,46	**0,167**
**Tempo de início da TARV^ǁ^ após diagnóstico**				
Variável contínua (em dias)	164,30(352,50)^⁋^	117,16(232,74)^⁋^	0,99	0,99-1,00	0,357
**Regime inicial da TARV^ǁ^**					
3TC+TDF+DTG	57(75,00)	63(82,89)	Referência		
3TC+TDF+EFZ	14(18,42)	8(10,53)	0,54	0,21-1,39	**0,203**
Outro	5(6,58)	5(6,58)	1,18	0,30-4,64	0,805
**Efeito colateral da TARV^ǁ^**					
Não	60(78,95)	66(86,84)	Referência		
Sim	16(21,05)	10(13,16)	0,56	0,22-1,38	**0,209**
**Número de esquemas realizados**					
1	62(81,58)	67(88,16)	Referência		
Mais que 1	14(18,42)	9(11,84)	0,58	0,22-1,51	0,270
Estadiamento do HIV^**^ segundo a OMS^††/ǁǁ^					
Estágio I	41(53,95)	38(50,00)	Referência		
Estágio II	16(21,05)	24(31,58)	1,66	0,76-3,61	**0,198**
Estágio III	11(14,47)	7(9,21)	0,70	0,24-2,00	0,513
Estágio IV	7(9,21)	5(6,58)	0,79	0,23-2,71	0,710
Ignorado	1(1,32)	2(2,63)			
**Primeiro resultado de CV^‡‡/ǁǁ^**					
Suprimida	59(77,63)	55(72,37)	Referência		
Não suprimida	9(11,84)	10(13,16)	1,21	0,45-3,21	0,696
Indetectável	8(10,53)	9(11,84)	1,40	0,48-4,30	0,528
Ignorado	0(0,00)	2(2,63)			
**Primeira contagem de CD4+^§§^**					
Variável contínua (em cel/mm^3^)	589,63(304,86) ^⁋^	600,81(285,01) ^⁋^	1,00	0,99-1,00	0,545
**Resultado de CV^‡‡^ antes da perda de seguimento^ǁǁ^**					
Suprimida	21(27,63)	18(23,68)	Referência		
Não suprimida	10(13,16)	7(9,21)	0,81	0,25-2,58	0,817
Indetectável	45(59,21)	49(64,47)	1,27	0,60-2,69	0,529
Ignorado	0(0,00)	2(2,63)			
**Contagem de CD4+^§§^ antes da perda de seguimento**					
Variável contínua (em cel/mm^3^)	701,47(340,39) ^⁋^	656,73(274,99) ^⁋^	1,00	0,99-1,00	0,449
**Infecção(ões) oportunista(s)^ǁǁ^**					
Não	12(15,79)	23(30,26)	Referência		
Sim	64(84,21)	52(68,42)	0,47	0,21-1,04	**0,065**
Ignorado	0(0,00)	1(1,32)			
**Infecção sexualmente transmissível^ǁǁ^**					
Não	47(61,84)	47(61,84)	Referência		
Sim	29(38,16)	28(36,84)	0,93	0,47-1,81	0,833
Ignorado	0(0,00)	1(1,32)			
**Falta(s) em consulta antes da perda de seguimento**					
Não	14(18,42)	26(34,21)	Referência		
Sim	62(81,58)	50(65,79)	0,44	0,20-0,93	**0,034**

*OR = O*dds ratio;* ^*†*^ IC95% = Intervalo de confiança de 95%; ^‡^p-valor referente ao teste qui-quadrado de Wald; ^§^CTA = Centro de Tratamento e Aconselhamento; ^ǁ^TARV = Terapia antirretroviral; ^⁋^Média (desvio-padrão); **HIV = Vírus da Imunodeficiência Humana; ^††^OMS = Organização Mundial da Saúde; ^‡‡^CV = Carga viral; ^§§^CD4+ = Células do sistema imunológico (linfócitos); ^ǁǁ^Ignorados excluídos da análise bivariada

O modelo final de regressão logística apresentou nível de significância com p-valor <0,05 com evidência estatística de associação para o grupo caso, sendo composto por sete variáveis. A razão de chance para retratamento entre adolescentes e jovens vivendo com HIV é de 3,46 vezes maior no grupo que não tem religião, quando comparado ao grupo que tem religião (IC95%: 1,52-7,88; p= 0,003). Já os fatores que diminuíram as chances de retratamento foram: idade de 21,91 anos no início do acompanhamento (ORaj: 0,78; IC95%: 0,62-0,98; p= 0,039), ser do sexo feminino (ORaj: 0,12; IC95%: 0,03-0,45; p= 0,001), morar em instituição (ORaj: 0,72; IC95%: 0,12-0,43; p= 0,004), uso de álcool (ORaj: 0,20; IC95%: 0,08-0,49; p<0,001), apresentar diagnóstico de HIV no CTA (ORaj: 0,31; IC95%: 0,13-0,74; p= 0,008) e apresentar falta(s) em consulta antes da perda de seguimento (ORaj: 0,20; IC95%: 0,07-0,52; p= 0,001) (Tabela 3).

**Tabela 3:** Análise multivariada das características sociodemográficas, comportamentais, clínicas, imunológicas e laboratoriais de adolescentes e jovens vivendo com HIV associadas ao retratamento após perda de seguimento. Maringá, PR, Brasil, 2024

Variável	Análise multivariada (ajustada)			
	OR*	IC95%^†^	p-valor^‡^
**Idade no início do acompanhamento (em anos)**	0,78	0,62-0,98	**0,039**
**Sexo**			
Masculino	Referência			
Feminino	0,12	0,03-0,45	**0,001**
**Religião**			
Não	3,46	1,52-7,88	**0,003**
Sim	Referência			
**Moradia**			
Com familiares	Referência			
Com amigos(as)	0,70	0,28-1,77	0,464
Em instituição	0,72	0,12-0,43	**0,004**
Sozinho(a)	0,46	0,04-5,47	0,545
**Uso de álcool**			
Não	Referência			
Sim	0,20	0,08-0,49	**<0,001**
**Diagnóstico no CTA^§^**			
Não	Referência		
Sim	0,31	0,13-0,74	**0,008**
**Falta(s) em consulta antes da perda de seguimento**			
Não	Referência			
Sim	0,20	0,07-0,52	**0,001**

*OR = O*dds ratio;* ^*†*^ IC95% = Intervalo de confiança de 95%; ^‡^p-valor referente ao teste qui-quadrado de Wald; ^§^CTA = Centro de Tratamento e Aconselhamento

Para este estudo, a AUC foi 0,82 (IC95%: 0,75-0,88), indicando que o modelo apresenta um bom desempenho. Além disso, o modelo preditivo final apresentou alta sensibilidade (81,6%) e especificidade (70,7%). 

## Discussão

Dentre os fatores associados ao retratamento entre adolescentes e jovens vivendo com HIV, foi evidenciado que a variável não ter religião apresenta associação estatisticamente significativa com retorno dos cuidados de HIV. Já as variáveis de idade no início do acompanhamento, sexo feminino, uso de álcool, morar em uma instituição, diagnóstico de HIV no CTA e faltas em consultas anteriores ao abandono associam-se estatisticamente à menor probabilidade de retorno ao tratamento.

Os principais resultados deste estudo sinalizam para a relevância de detectar os fatores associados para retratamento após perda de seguimento, os intrinsecamente relacionados ao contexto de vida de cada adolescente e jovem, assim como o de tratamento. Ainda há poucos estudos internacionais que abordam essa temática atualmente, tendo em vista a lacuna do conhecimento em relação às pesquisas voltadas ao retorno dos cuidados com HIV e sua relevância para populações prioritárias, como adolescentes e jovens^8^ ^-^ ^9^ ^,^ ^15^.

O retratamento após perda de seguimento é uma etapa crítica da cascata de tratamento do HIV, sendo necessário ser pesquisado para o alcance do paciente após qualquer falha no tratamento e assim poder facilitar o retorno^9^. Somado a isso, as mudanças demográficas e o aumento previsto do número de jovens vivendo com HIV ao longo dos próximos 20 anos sublinham a urgência de desenvolver modelos escaláveis de prestação de serviços de prevenção do HIV juntamente com o tratamento^3^.

Notou-se que não ter religião aumentou as chances de retorno ao tratamento; no entanto, a interpretação deve ser feita com cautela, pois religião é uma prática ou crença de fé organizada e/ou compartilhada. Enquanto espiritualidade refere-se à maneira como os indivíduos se relacionam com o transcendente^16^. A relação entre religião, estigma e HIV apresenta-se de maneira ambígua, conforme evidenciado em diferentes contextos culturais^17^ ^-^ ^18^.

Um estudo realizado na Indonésia identificou que discursos religiosos conservadores frequentemente reforçam o estigma ao associarem o HIV a comportamentos considerados moralmente condenáveis, como a homossexualidade, o uso de drogas e a multiplicidade de parceiros sexuais, gerando vergonha, culpa e estigma^17^. Além disso, a afiliação religiosa influencia a intensidade do estigma; assim, indivíduos de tradições conservadoras apresentaram maior propensão a estigmatizar pessoas vivendo com HIV em comparação a outras religiões^18^. 

Ressalta-se que ainda há uma associação entre HIV e punição divina, consequentemente, intensificando as atitudes discriminatórias e comprometendo a adesão ao tratamento. Assim, a religião se configura como um fator de duplo efeito - podendo reforçar preconceitos quando orientada por concepções moralistas ou contribuir para a redução do estigma e fortalecimento do cuidado quando pautada em discursos de inclusão e solidariedade^17^ ^-^ ^18^.

Uma possível explicação é que pessoas sem religião tendem a confiar mais na medicina e, por isso, podem manter ou retomar o tratamento. No entanto, o desejo de colocar a vida sob os princípios da religiosidade pode ter se originado na crença de que se poderia sair de uma condição de saúde difícil, como o HIV^16^. 

Estudo realizado em Zimbábue com jovens vivendo com HIV mostrou que colocar a vida sob a direção de Deus mediante a religião estava significativamente ligado ao aumento do fracasso do tratamento^16^. 

Além disso, outra explicação é o fato de que viver com HIV muitas vezes é associado a comportamentos pecaminosos e os jovens que viviam com HIV, por não serem considerados inocentes, também não eram dignos de proteção (pelas autoridades da igreja)^16^, fazendo com que eles não tivessem uma religião e buscassem o tratamento.

Dos fatores analisados quanto à sua influência negativa para o retratamento, a idade no início do acompanhamento demonstrou ser um fator que diminui as chances de retorno. Entretanto, torna-se necessária uma verificação mais aprofundada dos determinantes potenciais adicionais, uma vez que, durante a juventude, é importante o apoio psicossocial, dos familiares e das intervenções educacionais, fazendo com que tenham uma compreensão mais holística dos comportamentos de adesão ao tratamento^19^. 

Além disso, os adolescentes e jovens vivendo com HIV são influenciados por condições externas intimamente associadas a eles, como o esquecimento e problemas de saúde mental, contribuindo para a adesão abaixo do ideal^20^.

Notou-se que ser do sexo feminino diminuiu as chances do paciente adolescente e jovem vivendo com HIV retornar ao tratamento. A diferença de sexo pode estar relacionada ao fato de que as mulheres acessam mais serviços de teste de HIV do que homens, especialmente durante o pré-natal^11^ ^,^ ^21^ *.* Destacam-se as mulheres que engravidam, aderem ao tratamento pelo fato de quererem proteger o bebê. Estudo realizado no Sul do Brasil identificou que estar grávida aumentou as chances de adesão ao tratamento do HIV^22^ ^-^ ^23^.

Já no que se refere ao fato de as mulheres não retornarem ao tratamento, pode ser pelos desafios de serem as principais cuidadoras das crianças e dos demais membros da família, principalmente diante do adoecimento. E o fato de cuidarem da saúde dos familiares muitas vezes interfere no comparecimento às consultas e rotina com a TARV^11^ ^,^ ^21^ *.*

A questão de as mulheres não terem um trabalho remunerado também pode influenciá-las a não comparecer às consultas e ser retiradas da TARV^21^. Além disso, muitas mulheres têm dificuldade em revelar o seu estado sorológico, consequentemente, precisam se esconder enquanto tomam a TARV e, dessa forma, acabam esquecendo de tomar a medicação^22^.

Destaca-se que as desigualdades de sexo impactam diretamente no acesso e na permanência das mulheres no tratamento. Além disso, enfrentam a sobrecarga de papéis sociais, a dificuldade de negociação sexual, a dependência econômica e o estigma moral associado à soropositividade feminina; consequentemente, observam-se dificuldades não apenas na adesão inicial, mas também no retratamento. Logo, a feminização do HIV evidencia não só o aumento de casos entre mulheres, mas também as barreiras simbólicas e estruturais que comprometem sua continuidade no cuidado^24^ ^-^ ^25^.

O fato de morar em uma instituição para o tratamento de drogas e/ou detidos também faz com que haja uma barreira na conexão com o tratamento do HIV devido à falta de moradia própria. Dessa forma, torna-se necessário aumentar o engajamento no cuidado entre pessoas vivendo com HIV, revisando os modelos de prestação de serviços e reforçando o alcance da prestação de cuidados, abordando tanto a moradia quanto a redução de danos e o tratamento de transtornos por uso de substâncias, assim como intervenções específicas de sexo e gênero^25^.

Estudo realizado no Canadá mostra que a falta de moradia foi associada a uma redução de 44% nas chances de progressão geral pela cascata de cuidados do HIV, com uma redução de 41% nas chances de receber a TARV e redução de 54% em manter adesão e atingir a supressão da carga viral. Diante disso, reforça-se a necessidade de integração dos serviços para abordar os desafios interseccionais do HIV entre populações mais vulneráveis^26^.

Além disso, é importante incentivar a criação de uma rede de apoio consolidada às PVHIV para que, assim, possam tornar-se mais perseverantes, confiantes e aderir melhor ao tratamento^27^. 

A criação de apoio social entre familiares e amigos, principalmente quando o preconceito e o estigma da comunidade são fortes, torna-se uma estratégia poderosa para a normalização social^28^ ^-^ ^29^. Logo, os profissionais de saúde devem incentivar seus pacientes a criarem novos relacionamentos sociais tanto com aqueles que aceitam o diagnóstico de HIV quanto com outras PVHIV^28^. Dessa forma, o enfermeiro, durante a consulta de enfermagem no serviço especializado, deve identificar e mapear qual a rede de apoio do paciente presente no momento, com a finalidade de elaborar um plano de cuidado e incluí-los nele^27^.

O uso de álcool também foi um fator que prejudicou o retorno aos cuidados com HIV, por conta do comprometimento da memória prospectiva e do comportamento interativo de crenças de toxicidade/evitação devido à deterioração da doença física e do comportamento social. Logo, desenvolve-se dependência ao álcool, cuja abstinência causa efeitos adversos e, dessa forma, pode acarretar a não adesão intencional e não intencional^30^ ^-^ ^31^. 

Outra situação que pode interferir no retratamento é a estigmatização do uso de álcool entre PVHIV que estão em uso de TARV, ou seja, a sociedade tem a percepção de que o uso de álcool é vergonhoso e irresponsável devido aos comportamentos do indivíduo; assim, agravam-se os efeitos negativos do uso de álcool na adesão ao tratamento, comprometendo a eficácia do tratamento e a progressão da doença^32^ ^-^ ^33^. 

Dessa forma, os serviços de saúde devem estar atentos ao abuso de álcool entre as PVHIV, ainda mais adolescentes e jovens, que, diante da característica da própria idade, já se submetem a comportamentos de risco em saúde. Desta forma, torna-se necessário que os cuidados sejam integrados e que possam rastrear, tratar e monitorar o abuso de álcool e o do HIV, consequentemente, reduzindo a lacuna no tratamento e os resultados ruins do uso dessa substância em todas as etapas de cuidados do HIV^32^ ^-^ ^33^.

O diagnóstico no CTA também influencia o retorno dos cuidados de HIV e, ainda que o respectivo serviço em que foi realizado este estudo tenha o aconselhamento, os próprios adolescentes e jovens têm que lidar com o estigma que compromete a capacidade de tomar a TARV abertamente e sem medo. E com a adesão comprometida, a carga viral não suprimida é quase inevitável. Assim, o aconselhamento deve destacar a natureza crônica da doença e a necessidade de tratar com TARV por toda a vida^34^.

Um estudo realizado em Uganda mostra que a boa adesão à TARV é crucial para a supressão da CV, mas muitos jovens desconhecem esse fato**.** Além disso, os adolescentes que nunca atingiram uma CV suprimida após o início do tratamento expressaram preocupações sobre não saber a justificativa para tomar pílulas com periodicidade^34^; assim, esse contexto também pode influenciar o retratamento.

A falta em consultas antes do abandono é um fator que diminui as chances de retratamento entre adolescentes e jovens vivendo com HIV, e os pacientes que apresentam esse comportamento são considerados desviantes ou não cumpridores do tratamento pelos profissionais de saúde e pelo sistema de saúde geral^21^.

Os adolescentes e jovens vivendo com HIV necessitam de uma vida inteira de tratamento para o HIV, sendo cruciais consultas médicas, coletas de sangue regulares e medicamentos desagradáveis^35^. E somado a isso, esses pacientes ainda lidam com os contextos físicos, emocionais e sociais, afetando a tomada de decisões e os comportamentos relacionados ao acompanhamento e à adesão ao tratamento. Além disso, a perda de seguimento também piora do início da adolescência para o final da juventude/início da idade adulta, possivelmente por conta do menor envolvimento dos responsáveis e do aumento da responsabilidade e autonomia^35^.

Uma estratégia para reduzir a falta nas consultas e retiradas da TARV são as prescrições para vários meses (3 ou 6 meses) ou o uso de novas tecnologias, como a TARV injetável de longa duração, que, portanto, podem ajudar o jovem a conciliar as prioridades dos cuidados de HIV e a vida fora do serviço especializado^21^.

Além disso, as estratégias adicionais para melhorar a resiliência e o alcance do paciente após qualquer lacuna no tratamento podem facilitar o retorno, assim como aumentar a conscientização dos gestores e profissionais de saúde para reconhecer os esforços dos pacientes para permanecerem no acompanhamento^21^ ^,^ ^36^.

Referente à limitação do estudo, aponta-se o uso exclusivo de dados secundários a partir do prontuário dos adolescentes e jovens, os quais podem apresentar incompletude de informação e, portanto, viés de interpretação. 

As implicações para o avanço do conhecimento científico para a área de saúde e enfermagem estão relacionadas ao desenvolvimento de um estudo que responde a uma lacuna do conhecimento voltada a este público. Os resultados permitem que os enfermeiros e demais profissionais de saúde elaborem um plano de cuidado baseado na necessidade do paciente adolescente e jovem com a finalidade de prevenir o abandono permanente do tratamento. Sugerem-se futuros estudos sobre o reengajamento, ou seja, o retorno ao tratamento dos adolescentes e jovens vivendo com HIV voltado às medidas desses preditores para pesquisar seus mecanismos de efeito causal no retratamento. 

## Conclusão

O retorno ao tratamento por adolescentes e jovens vivendo com HIV tem determinantes multifatoriais associados ao perfil sociodemográfico, comportamental e clínico. 

Notou-se que não ter prática religiosa interfere no retratamento e os fatores negativamente associados ao retorno desse público ao serviço foram ser do sexo feminino, fazer uso de álcool, morar em instituição, diagnóstico no CTA e apresentar falta(s) em consultas antes da perda de seguimento. Esses resultados fornecem *insights* sobre a necessidade de rever as práticas em saúde para que haja prevenção do abandono permanente e promoção a retenção dos adolescentes e jovens no tratamento de HIV. Além disso, destaca-se a necessidade de ações práticas, como o fortalecimento de políticas públicas externas voltadas à moradia, maior inclusão de mulheres (não apenas gestantes) e redução de preconceitos em estratégias de prevenção, tratamento e cuidado com o HIV entre os adolescentes e jovens. 

## Data Availability

Todos os dados gerados ou analisados durante este estudo estão incluídos neste artigo publicado.

# Translation: Retratamiento tras pérdida de seguimiento en pacientes adolescentes y jóvenes que viven con el VIH: estudio de casos y controles^*^

**DOI:** 10.1590/1518-8345.7973.4800

**Authors:** Camila Moraes Garollo Piran, Alana Vitória Escritori Cargnin, Mariana Martire Mori, Rosana Rosseto de Oliveira, João Manuel Graça Frade, Marcela Demitto Furtado

**Affiliations:** 1 Universidade Estadual de Maringá, Maringá, PR, Brasil.; 2 Polytechnic University of Leiria, Leiria, Portugal.; 3 Scholarship holder at the Coordenação de Aperfeiçoamento de Pessoal de Nível Superior (CAPES), Brasil.

**Keywords:** Retratamiento, Perdida de Seguimiento, Adolescentes, Adulto Joven, VIH, Síndrome de Inmunodeficiencia Adquirida.

## Abstract

**Objetivo:**

identificar los factores asociados al retratamiento después de la pérdida de seguimiento en adolescentes y jóvenes que viven con el Virus de la Inmunodeficiencia Humana.

**Método:**

estudio de casos y controles pareados (razón 1/1) con recolección de datos de historias clínicas. Los casos fueron adolescentes y jóvenes con diagnóstico de Virus de Inmunodeficiencia Humana/Síndrome de Inmunodeficiencia Adquirida con retratamiento después de la pérdida de seguimiento. Los controles fueron adolescentes y jóvenes diagnosticados, perdidos en el seguimiento, sin antecedentes de retratamiento. Para el análisis estadístico, se adoptó la regresión logística.

**Resultados:**

se incluyeron 76 casos y 76 controles. La variable asociada con la mayor probabilidad de retratamiento fue no tener una religión (ORad:3,46; IC95%: 1,52-7,88; p=0,003). Los factores que disminuyeron las probabilidades de retratamiento fueron los 21,91 años de edad al inicio del seguimiento (ORaj:0,78; IC95%: 0,62-0,98; p=0,039), género femenino (ORaj:0,12; IC95%: 0,03-0,45; p=0,001), viviendo en una institución (ORaj:0,72; IC95%: 0,12-0,43; p=0,004), consumo de alcohol (ORaj: 0,20; IC95%: 0,08-0,49; p<0,001), diagnóstico del Virus de la Inmunodeficiencia Humana en el Centro de Tratamiento y Asesoramiento (ORaj:0,31; IC95%: 0,13-0,74; p=0,008) y las citas perdidas antes de la pérdida de seguimiento (ORaj: 0,20; IC95%: 0,07-0,52; p=0,001).

**Conclusión:**

el retorno al tratamiento de adolescentes y jóvenes que viven con el Virus de la Inmunodeficiencia Humana tiene determinantes multifactoriales asociados al perfil sociodemográfico, conductual y clínico.

## Destacados

**(1)** Primer estudio brasileño que aborda el retratamiento entre jóvenes que viven con el VIH. **(2)** La ausencia de afiliación religiosa se asoció con una mayor probabilidad de retratamiento. **(3)** El sexo, el consumo de alcohol y la convivencia en instituciones se asociaron con el retratamiento. **(4)** El diagnóstico en la CTA y las citas perdidas disminuyeron las posibilidades de retratamiento. **(5)** Proporciona información sobre la prevención del abandono permanente.

## Introducción

Se estima que, en 2023, había 40 millones de personas viviendo con el Virus de Inmunodeficiencia Humana (VIH) (PVVIH), y dos de cada siete nuevas infecciones por VIH se producen entre adolescentes y jóvenes^1^. Incluso con la terapia antirretroviral (TAR) que tiene un impacto relevante en la reducción de la morbilidad y mortalidad entre las personas que viven con el VIH, los adolescentes y jóvenes están más expuestos a contextos negativos^2^, ya que este grupo de edad suele estar ausente en la cascada de tratamiento del VIH^3^.

La inclusión del TAR ha llevado a que el VIH se convierta en una condición de salud crónica controlable, proporcionando una mejor calidad de vida, un retorno a la esperanza de vida normal y un impacto sustancial en la prevención y el tratamiento del VIH. Sin embargo, para los adolescentes y jóvenes que viven con esta condición, todavía es difícil mantener la adhesión al tratamiento debido al uso diario de TAR a lo largo de la vida^4^ ^-^ ^6^.

La falta de adhesión continua o la pérdida de seguimiento provoca interrupciones prolongadas del tratamiento o interrupción del TAR, lo que constituye una de las principales barreras para la terminación del SIDA (Síndrome de Inmunodeficiencia Adquirida), ya que puede conducir a la resistencia a los medicamentos y, en consecuencia, al fracaso del tratamiento^5^ ^-^ ^6^. Además, los adolescentes y jóvenes que viven con el VIH tienen peores resultados en relación al tratamiento del VIH en comparación con otros grupos de edad que viven con el VIH, debido a las conductas de riesgo para la salud que presentan en este grupo de edad^5^.

Dicho esto, se percibe que muchos adolescentes y jóvenes que viven con VIH abandonan el tratamiento^7^ y, después de un cierto período, regresan al servicio^8^. El retorno a la atención del VIH tras la pérdida de seguimiento es un comportamiento frecuente, aunque poco comprendido. El retratamiento está cada vez más presente en la cascada de atención del VIH, en la que muchos individuos entran y salen del cuidado después de iniciar el tratamiento y a lo largo de la vida^8^ ^-^ ^9^.

A pesar de que se han estudiado los factores que impulsan la pérdida de seguimiento o el abandono^7^ ^,^ ^10^ ^-^ ^11^, se sabe poco sobre los factores que facilitan y dificultan el retorno al tratamiento^8^ ^-^ ^9^. Los resultados de este público específico aún son incipientes y, como consecuencia, imposibilitan que los profesionales de la salud, como los enfermeros que están en la gestión del servicio, identifiquen los factores asociados al retratamiento e implementen estrategias preventivas para evitar el abandono permanente^8^.

Explorar el retratamiento después de la pérdida durante el seguimiento en un entorno que brinda tratamiento en diferentes entornos puede contribuir a la implementación de nuevas estrategias para mejorar los resultados entre adolescentes y jóvenes. Esto será crucial para alcanzar los objetivos mundiales de eliminación del SIDA para 2030. Así, este estudio tiene como objetivo responder a la siguiente pregunta de investigación: ¿cuáles son los factores asociados con el retorno de la atención del VIH entre adolescentes y jóvenes? Dicho esto, el objetivo de este estudio fue identificar los factores asociados con el retratamiento después de la pérdida de seguimiento entre adolescentes y jóvenes que viven con el VIH.

## Método

### Tipo de estudio

Estudio epidemiológico, del tipo caso-control emparejado con una proporción de 1 caso/1 control de adolescentes y jóvenes que viven con el VIH, que se integró en una cohorte ambispectiva. La proporción de 1:1 se estableció en función del tiempo disponible para la realización de esta investigación. Se utilizó el *Strengthening the Reporting of Observational Studies in Epidemiology* (STROBE) para informar sobre el estudio, tal y como recomienda la *Enhancing the Quality and Transparency of Health Research* (EQUATOR *Network*)^12^.

### Escenario del estudio

El escenario del estudio fue el Servicio de Atención Especializada (SAE) perteneciente a la Clínica Ambulatoria de Infecciones de Transmisión Sexual/VIH/SIDA del 15º Distrito Regional de Salud, ubicada en el noroeste del Estado de Paraná, Brasil. Es un servicio público, especializado y de referencia que presta servicio a Maringá y a otros 29 municipios. 

### Población

Los adolescentes y jóvenes diagnosticados con VIH/SIDA se definieron como casos, de acuerdo con la Clasificación Internacional de Enfermedades (CIE-10), un indicador de estadísticas de problemas de mortalidad y morbilidad, representados por los códigos B20.0 a B24, con un retratamiento posterior después de la pérdida durante el seguimiento. Mientras que los controles fueron adolescentes y jóvenes con CIE-10 relacionados con VIH/SIDA, que se perdieron durante el seguimiento en el NCS después de iniciar el TAR, sin antecedentes de retratamiento.

Se consideraron los siguientes criterios de inclusión: tener entre 10 y 24 años, haber adquirido el VIH por transmisión sexual y haber iniciado el TAR. Se destaca que el grupo de edad de 10 a 19 años y de 20 a 24 años corresponde al período de adolescencia y juventud, respectivamente^13^. Los siguientes criterios de no inclusión se definieron como pacientes que no iniciaron TAR y aquellos que adquirieron el VIH por transmisión vertical. Y los criterios de exclusión fueron: pacientes que fallecieron y cuando no hubo caso ni control para emparejamiento.

### Definición de la muestra

Se utilizó la muestra total de 198 adolescentes y jóvenes que viven con VIH que se encontraban en situación de pérdida de seguimiento, atendidos en el servicio entre enero de 2017 y diciembre de 2023. Después del análisis exploratorio de las historias clínicas de pacientes adolescentes y jóvenes, verificando los criterios de selección mencionados anteriormente, se realizó el emparejamiento muestral, buscando la mayor similitud posible entre los individuos.

Los grupos fueron emparejados por grupo de edad y año de ingreso al servicio. La variable grupo de edad se estableció en el emparejamiento para proporcionar una mayor similitud entre las edades de los casos y los controles; Aunque algunas edades no eran exactamente iguales entre algunas parejas, todavía estaban cerca. No se incluyeron 19 pacientes que no iniciaron TAR y cinco pacientes que adquirieron el VIH por transmisión vertical. Se excluyeron tres muertes y 19 pacientes que fueron casos debido a la falta de controles dentro del período de estudio (Figura 1).

**Figura 1:** Selección de las historias clínicas de adolescentes y jóvenes que viven con el VIH. Maringá, PR, Brasil, 2024

### Variables del estudio

Las informaciones de los pacientes con respecto a cada variable se recopilaron de los registros de salud. La variable dependiente (retratamiento después de la pérdida de seguimiento) se adoptó para la regresión logística con el fin de verificar la asociación con las variables independientes.

*Variable dependiente*

Se adoptó como variable dependiente (resultado) el reingreso tras la pérdida de seguimiento. El retratamiento o regreso a la atención se define como el tratamiento del paciente después del abandono, con una repetición del mismo tratamiento inicial o con una medida adicional o alternativa. El retratamiento se utiliza a menudo con referencia a una modalidad diferente, cuando el original se utilizó de manera inapropiada, nociva o no tuvo éxito^8^ ^-^ ^9^ ^,^ ^14^.

*Variables independientes*

Como variables independientes, se agruparon las siguientes características:

- Sociodemográficas: edad al diagnóstico (en años), edad al inicio del seguimiento (en años), sexo (hombre; mujer), estado civil (sin pareja; con pareja), raza/color (blanco; no blanco), educación (<12 años; ≥12 años), religión (no; sí), orientación sexual (heterosexual; homosexual/bisexual), ocupación (empleado/autónomo; estudiante/ama de casa; desempleado), vivienda (con familia; con amigos; en una institución; solo; ignorado);
- Comportamentales: consumo de alcohol (no; sí), consumo de tabaco (no; sí), consumo de drogas (no; sí), pareja sexual (fijo; casual), comorbilidad(es) (no; sí), trastorno(s) mental(es) (no; sí);
- Clínicos, inmunológicos y de laboratorio: diagnóstico en el Centro de Tratamiento y Asesoramiento (CTA) (no; sí), tipo de ingreso (caso nuevo; caso transferido), tiempo de inicio del TAR después del diagnóstico (en días), régimen inicial de TAR [Lamivudina (3TC) + Tenofovir (TDF) + Dolutegravir (DTG); Lamivudina (3TC) + Tenofovir (TDF) + Efavirenz (EFV); Otros], efecto secundario del TAR (no; sí), número de regímenes realizados (1; más de 1), estadificación del VIH en la OMS (estadio I; estadio II; estadio III; estadio IV; desconocido), primer resultado de la carga viral (CV) (suprimido; no suprimido; indetectable; desconocido), primer recuento de linfocitos T auxiliares CD4+ (CD4+) (en células/mm^3^), resultado de la CV antes de la pérdida durante el seguimiento (suprimido; no suprimido; indetectable; desconocido), recuento de CD4+ antes de la pérdida durante el seguimiento (en célula/mm^3^), infección(es) oportunista(s) (no; sí; desconocida), otra infección de transmisión sexual (no; sí; desconocida), consulta perdida antes de la pérdida de seguimiento (no; sí);

### Instrumentos utilizados para la recopilación de información

Los datos se recopilaron de fuentes secundarias utilizando los registros de salud disponibles en la clínica ambulatoria de Infecciones de Transmisión Sexual/VIH/SIDA. El instrumento de recolección de datos se estableció según lo recomendado por el Ministerio de Salud, y está de acuerdo con el formulario de notificación de casos de VIH y seguimiento ambulatorio. Para garantizar la comparabilidad de los datos, se utilizó el mismo instrumento para los casos y los controles.

### Recopilación de datos

Los datos se recopilaron entre junio y octubre de 2024, con dos asistentes de investigación, uno estudiante de maestría y el otro estudiante de doctorado, ambos participantes en el proyecto de investigación. Los asistentes de investigación fueron capacitados *in loco* por el investigador principal y ayudaron en la recopilación y doble verificación de datos para garantizar la veracidad de la información. Ambos estaban cegados a los objetivos de la investigación, así como a la pregunta del estudio, como una estrategia para minimizar el sesgo de confusión, en la posibilidad de establecer alguna relación entre las respuestas. La información fue ingresada y almacenada en una base de datos administrada por el investigador principal. Además, del emparejamiento, la variable de registro y la variable de número de registro médico se utilizaron como información adicional para diferenciar mejor los registros médicos y minimizar el sesgo de selección.

### Tratamiento y análisis de datos

Para el tratamiento de los datos se utilizó *Microsoft Excel* ^*®*^ y luego los datos fueron transportados al *software SPSS Statistics* ^*®*^ , versión 25.0 para el análisis de los datos. Inicialmente, se describieron las características sociodemográficas, conductuales, clínicas, inmunológicas y de laboratorio de adolescentes y jóvenes que viven con VIH, según el resultado de retratamiento o no después de la pérdida al seguimiento, utilizando medidas de frecuencia absoluta y relativa, tendencia central (media) y dispersión (desviación estándar). A continuación, para verificar los factores asociados al reingreso al tratamiento en adolescentes y jóvenes que habían interrumpido el tratamiento, se utilizaron modelos de regresión logística binomial.

Se realizaron análisis bivariados para comparar cada variable con el resultado en estudio. Aquellos con un valor de p ≤0,25 en la prueba de Wald de chi-cuadrado se evaluaron en relación con la multicolinealidad, de modo que se pudieron construir modelos multivariados parsimoniosos y robustos. Luego, se probaron múltiples modelos utilizando la metodología de selección de variables hacia atrás por pasos. Las variables independientes con un valor de p de <0,05 en la prueba se conservaron en los modelos finales, cuando se ajustaron entre sí.

Como medida de asociación, se calcularon los *odds ratios* (OR) y los *odds ratios* ajustados (ORaj) seguidos de sus intervalos de confianza del 95% (IC95%). Estas razones representaron el aumento o la reducción de la probabilidad de cada subvariable para la ocurrencia de un nuevo tratamiento después de la pérdida de seguimiento en relación con las subcategorías de referencia en las respectivas variables independientes. El modelo final se consideró significativo en comparación con el nulo en la prueba ómnibus.

Para el modelo múltiple final, se realizó un análisis de las características operativas del receptor (ROC), una forma útil de evaluar la precisión de las predicciones del modelo. A partir de esto, se calculó el área bajo la curva (AUC), que representa la probabilidad de que la predicción esté en el orden correcto al observar una variable de prueba. Además, la sensibilidad y especificidad del modelo final se calcularon en el punto de corte ideal, definido por el índice de Youden.

### Aspectos éticos

Aunque no hubo contacto directo con los participantes, las historias clínicas contenían información confidencial. Así, en cumplimiento de las Resoluciones N.º 466/12 y 510/16 del Consejo Nacional de Salud (CNS), el estudio fue sometido al Comité Permanente de Ética en la Investigación con Seres Humanos de la institución firmante, siendo aprobado bajo el parecer N.º 5.202.623/2022 y N.º 6.897.755/2024, Certificado de Presentación de Apreciación Ética (CAAE): 52331221.3.0000.0104.

## Resultados

Se analizaron un total de 198 historias clínicas de adolescentes y jóvenes que viven con VIH, y 152 fueron elegibles para el estudio, correspondientes a 76 casos (retratamiento) y 76 controles (no retratamiento). En cuanto a las características sociodemográficas y conductuales de los adolescentes y jóvenes de los casos y controles, la edad media al diagnóstico fue de 21,39 años y 21,65 años, respectivamente. En ambos grupos, la mayoría eran hombres y sin pareja.

Se identificó una asociación estadísticamente significativa entre el retratamiento y las variables edad al inicio del seguimiento (p=0,062), sexo (p=0,010), raza/color (p=0,233), religión (p=0,003), ocupación (p=0,114), vivienda (p=0,032) y consumo de alcohol (p=0,022). La probabilidad de retratamiento después de la pérdida de seguimiento fue mayor entre los individuos de raza/color no blancos (OR: 1,55; IC95%: 0,75-3,18), los que no tienen religión (OR: 2,70; IC95%: 1,39-5,25), que están desempleados (OR: 3,04; IC95%: 0,76-12,12) y viven en una institución (OR: 0,17; IC95%: 0,03-0,86) (Tabla 1).

**Tabla 1:** Análisis descriptivo y bivariado de las características sociodemográficas y conductuales de adolescentes y jóvenes que viven con VIH, según el resultado del retratamiento o no después de la pérdida de seguimiento. Maringá, PR, Brasil 2024

Variable	Caso	Control	Análisis bivariado (crudo)		
	n(%)	n(%)	OR*	IC95%^†^	valor p^‡^
**Edad en el momento del diagnóstico**				
Variable continua (en años)	21,39(1,96)^§^	21,65(2,00)^§^	1,05	0,89-1,25	0,413
**Edad al inicio del seguimiento**				
Variable continua (en años)	21,91(1,84)^§^	22,47(1,79)^§^	0,84	0,98-1,42	**0,062**
**Sexo**					
Masculino	56(73,68)	69(90,79)	Referencia		
Femenino	20(26,32)	7(15,79)	0,29	0,11-0,74	**0,010**
**Estado civil**					
Sin pareja	60(78,95)	65(85,53)	Referencia		
Con pareja	16(21,05)	11(14,47)	0,65	0,28-1,52	0,326
**Raza/color**					
Blanco	58(76,32)	51(67,11)	Referencia		
No blanco	18(23,68)	25(32,89)	1,55	0,75-3,18	**0,233**
**Educación**					
<12 años	26(34,21)	24(31,58)	Referencia		
≥12 años	50(65,79)	52(68,42)	1,08	0,54-2,14	0,818
**Religión**					
No	29(38,16)	48(63,16)	2,70	1,39-5,25	**0,003**
Sí	47(61,84)	28(36,84)	Referencia		
**Orientación sexual**					
Heterosexual	23(30,26)	17(22,37)	Referencia		
Homosexual/bisexual	53(69,74)	59(77,63)	1,45	0,70-3,02	0,313
**Ocupación**					
Empleado/autónomo	57(75,00)	50(65,79)	Referencia		
Estudiante/ama de casa	16(21,05)	18(23,68)	1,14	0,51-2,52	0,741
Desempleado	3(3,95)	8(10,53)	3,04	0,76-12,12	**0,114**
**Vivienda^||^**					
Con miembros de la familia	49(64,47)	36(10,53)	Referencia		
Con amigos(as)	2(2,63)	12(15,79)	1,42	0,69-2,90	0,337
En la institución	2(2,63)	3(3,95)	0,17	0,03-0,86	**0,032**
Solo(a)	23(30,26)	24(31,58)	0,69	0,01-4,55	0,705
Ignorado	0(0,00)	1(1,32)			
**Consumo de alcohol**					
No	25(32,89)	39(51,32)	Referencia		
Sí	51(67,11)	37(48,68)	0,46	0,23-0,89	**0,022**
**Consumo de tabaco**					
No	46(60,53)	47(61,84)	Referencia		
Sí	30(39,47)	29(38,16)	0,88	0,45-1,70	0,706
**Consumo de drogas**					
No	52(68,42)	54(71,05)	Referencia		
Sí	24(31,58)	22(28,95)	0,85	0,42-1,73	0,669
**Pareja sexual**					
Fijo	39(51,32)	34(44,74)	Referencia		
Casual	37(48,68)	42(55,26)	1,38	0,72-2,65	0,321
**Comorbilidade(s)**					
No	71(93,42)	69(90,79)	Referencia		
Sí	5(6,58)	7(9,21)	1,48	0,44-4,91	0,517
**Trastorno(s) mental(es)**					
No	64(84,21)	61(80,26)	Referencia		
Sí	12(15,79)	15(19,74)	1,24	0,53-2,91	0,612

*OR = *Odds ratio* (Razón de probabilidades); ^†^IC95% = Intervalo de confianza del 95%; ^‡^Valor p correspondiente a la prueba chi-cuadrado de Wald; **§**Media (desviación estándar); ^||^Ignorados excluidos del análisis bivariado

En cuanto a las características clínicas, inmunológicas y de laboratorio de los adolescentes y jóvenes que viven con VIH, se observó que se trataba de casos nuevos, y el tiempo promedio de inicio del TAR después del diagnóstico entre casos y controles fue de 164,30 días y 117,16 días, respectivamente. Las variables asociadas al retratamiento fueron: diagnóstico en la ATC (p= 0,255), tipo de ingreso al servicio (p=0,167), régimen inicial de TAR (p=0,203), efecto secundario del TAR (p=0,209), estadificación del VIH después de la OMS (p=0,198), infecciones oportunistas (p=0,065) y faltas a las consultas antes de la pérdida de seguimiento (p=0,034). La razón de probabilidades para el retorno al tratamiento fue mayor entre los casos transferidos (OR: 1,67; IC95%: 0,80-3,46) y VIH estadio II según la OMS (OR: 1,66; IC95%: 0,76-3,61) en comparación con las otras categorías (Tabla 2).

El modelo de regresión logística final mostró un nivel de significancia de valor de p <0,05 con evidencia estadística de asociación para el grupo de casos, que consta de siete variables. La razón de probabilidades para el retratamiento entre adolescentes y jóvenes que viven con VIH es 3,46 veces mayor en el grupo que no tiene religión, en comparación con el grupo que tiene religión (IC95%: 1,52-7,88; p = 0,003). Los factores que disminuyeron las probabilidades de retratamiento fueron: edad de 21,91 años al inicio del seguimiento (ORaj: 0,78; IC95%: 0,62-0,98; p= 0,039), ser mujer (ORaj: 0,12; IC95%: 0,03-0,45; p= 0,001), viviendo en una institución (ORaj: 0,72; IC95%: 0,12-0,43; p= 0,004), consumo de alcohol (ORaj: 0,20; IC95%: 0,08-0,49; p<0,001), con diagnóstico de VIH en la CTA (ORaj 0,31; IC95%: 0,13-0,74; p= 0,008) y tener ausencia(s) de una consulta antes de la pérdida para el seguimiento (ORaj: 0,20; IC95%: 0,07-0,52; p= 0,001) (Tabla 3).

**Tabla 2:** Análisis descriptivo y bivariado de las características clínicas, inmunológicas y de laboratorio de adolescentes y jóvenes que viven con VIH, según el resultado de retratamiento o no después de la pérdida de seguimiento. Maringá, PR, Brasil 2024

Variable	Caso	Control	Análisis bivariado (crudo)		
	n(%)	n(%)	OR*	IC95%^†^	valor p^‡^
**Diagnóstico en CTA^§^**					
No	36(47,37)	31(40,79)		Referencia	
Sí	40(52,63)	45(59,21)	0,68	0,35-1,31	0,255
**Tipo de entrada**					
Nuevo caso	58(76,32)	51(67,11)	Referencia		
Caso transferido	18(23,68)	25(32,89)	1,67	0,80-3,46	**0,167**
**Tiempo para comenzar TAR^||^ después del diagnóstico**				
Variable continua (en días)	164,30(352,50) ^¶^	117,16(232,74) ^¶^	0,99	0,99-1,00	0,357
**Régimen inicial del TAR^||^**					
3TC+TDF+DTG	57(75,00)	63(82,89)	Referencia		
3TC+TDF+ EFV	14(18,42)	8(10,53)	0,54	0,21-1,39	**0,203**
Otro	5(6,58)	5(6,58)	1,18	0,30-4,64	0,805
**Efecto secundario del TAR^||^**					
No	60(78,95)	66(86,84)	Referencia		
Sí	16(21,05)	10(13,16)	0,56	0,22-1,38	**0,209**
**Número de esquemas realizados**					
1	62(81,58)	67(88,16)	Referencia		
Más de 1	14(18,42)	9(11,84)	0,58	0,22-1,51	0,270
Estadificación del VIH** según la OMS^††/||||^					
Estadio I	41(53,95)	38(50,00)	Referencia		
Estadio II	16(21,05)	24(31,58)	1,66	0,76-3,61	0,198
Estadio III	11(14,47)	7(9,21)	0,70	0,24-2,00	0,513
Estadio IV	7(9,21)	5(6,58)	0,79	0,23-2,71	0,710
Ignorado	1(1,32)	2(2,63)			
**Primer resultado del CV^‡‡/||||^**					
Suprimido	59(77,63)	55(72,37)	Referencia		
No suprimido	9(11,84)	10(13,16)	1,21	0,45-3,21	0,696
Indetectable	8(10,53)	9(11,84)	1,40	0,48-4,30	0,528
Ignorado	0(0,00)	2(2,63)			
**Primer recuento de CD4+^§§^**					
Variable continua (en cel/mm^3^)	589,63(304,86) ^¶^	600,81(285,01) ^¶^	1,00	0,99-1,00	0,545
**Resultado del CV^‡‡^ antes de la pérdida de seguimiento^||||^**					
Suprimido	21(27,63)	18(23,68)	Referencia		
No suprimido	10(13,16)	7(9,21)	0,81	0,25-2,58	0,817
Indetectable	45(59,21)	49(64,47)	1,27	0,60-2,69	0,529
Ignorado	0(0,00)	2(2,63)			
**Recuento de CD4+^§§^ antes de la pérdida de seguimiento**					
Variable continua (en cel/mm^3^)	701,47(340,39)^||^	656,73(274,99)^||^	1,00	0,99-1,00	0,449
**Infección(ones) oportunista(s)^||||^**					
No	12(15,79)	23(30,26)	Referencia		
Sí	64(84,21)	52(68,42)	0,47	0,21-1,04	**0,065**
Ignorado	0(0,00)	1(1,32)			
**Infección de transmisión sexual^||||^**					
No	47(61,84)	47(61,84)	Referencia		
Sí	29(38,16)	28(36,84)	0,93	0,47-1,81	0,833
Ignorado	0(0,00)	1(1,32)			
**Ausencia(s) en la consulta antes de la pérdida de seguimiento**					
No	14(18,42)	26(34,21)	Referencia		
Sí	62(81,58)	50(65,79)	0,44	0,20-0,93	**0,034**

*OR = *Odds ratio* (Razón de probabilidades); ^†^IC95% = Intervalo de confianza del 95%; ^‡^Valor p correspondiente a la prueba chi-cuadrado de Wald; ^§^CTA = Centro de Tratamiento y Asesoramiento; ^||^TAR = Terapia antirretroviral; ^¶^Media (desviación estándar); **VIH = Virus de la inmunodeficiencia humana; ^††^OMS = Organización Mundial de la Salud; ^‡‡^CV = Carga viral; ^§§^CD4+ = Células del sistema inmunitario (linfocitos); ^||||^Ignorados excluidos del análisis bivariado

**Tabla 3:** Análisis multivariado de las características sociodemográficas, conductuales, clínicas, inmunológicas y de laboratorio de adolescentes y jóvenes que viven con VIH asociadas al retratamiento después de la pérdida de seguimiento. Maringá, PR, Brasil 2024

Variable	Análisis multivariante (ajustado)		
	OR*	IC95%^†^	Valor p^‡^
**Edad al inicio del seguimiento (en años)**	0,78	0,62-0,98	**0,039**
**Sexo**			
Masculino	Referencia		
Femenino	0,12	0,03-0,45	**0,001**
**Religión**			
No	3,46	1,52-7,88	**0,003**
Sí	Referencia		
**Vivienda**			
Con miembros de la familia	Referencia		
Con amigos(as)	0,70	0,28-1,77	0,464
En la institución	0,72	0,12-0,43	**0,004**
Solo(a)	0,46	0,04-5,47	0,545
**Consumo de alcohol**			
No	Referencia		
Sí	0,20	0,08-0,49	**<0,001**
**Diagnóstico en CTA^§^**			
No	Referencia		
Sí	0,31	0,13-0,74	**0,008**
**Ausencia(s) en la consulta antes de la pérdida de seguimiento**			
No	Referencia		
Sí	0,20	0,07-0,52	**0,001**

*OR = Razón de probabilidades; ^†^IC95% = Intervalo de confianza del 95%; ^‡^valor p correspondiente a la prueba chi-cuadrado de Wald; ^§^CTA = Centro de Tratamiento y Asesoramiento

Para este estudio, el AUC fue de 0,82 (IC95%: 0,75-0,88), lo que indica que el modelo funciona bien. Además, el modelo predictivo final mostró alta sensibilidad (81,6%) y especificidad (70,7%).

## Discusión

Entre los factores asociados al retratamiento entre adolescentes y jóvenes que viven con VIH, se evidenció que la variable no tener religión tiene una asociación estadísticamente significativa con el retorno a la atención del VIH. Por otro lado, las variables de edad al inicio del seguimiento, sexo femenino, consumo de alcohol, vivir en una institución, diagnóstico de VIH en el CTA y faltar a las consultas antes del abandono se asociaron estadísticamente con una menor probabilidad de volver al tratamiento.

Los principales resultados de este estudio indican la relevancia de detectar los factores asociados al retratamiento después de la pérdida para el seguimiento, los intrínsecamente relacionados con el contexto de vida de cada adolescente y joven, así como el tratamiento. Todavía son pocos los estudios internacionales que abordan este tema en la actualidad, en vista de la brecha de conocimiento en relación con la investigación dirigida al retorno de la atención del VIH y su relevancia para poblaciones prioritarias, como adolescentes y jóvenes^8^ ^-^ ^9^ ^,^ ^15^.

El retratamiento después de la pérdida de seguimiento es una etapa crítica de la cascada de tratamiento del VIH, y necesita ser investigado para llegar al paciente después de cualquier fracaso del tratamiento y así facilitar el retorno^9^. Además, los cambios demográficos y el aumento esperado en el número de jóvenes que viven con el VIH en los próximos 20 años subrayan la urgencia de desarrollar modelos escalables de prestación de servicios de prevención del VIH junto con el tratamiento^3^.

Se observó que no tener una religión aumentaba las posibilidades de volver al tratamiento; Sin embargo, la interpretación debe hacerse con precaución, ya que la religión es una práctica o creencia de fe organizada y/o compartida. Mientras que la espiritualidad se refiere a la forma en que los individuos se relacionan con lo trascendente^16^. La relación entre religión, estigma y VIH es ambigua, como se evidencia en diferentes contextos culturales^17^ ^-^ ^18^.

Un estudio realizado en Indonesia identificó que los discursos religiosos conservadores a menudo refuerzan el estigma al asociar el VIH con comportamientos considerados moralmente reprobables, como la homosexualidad, el uso de drogas y la multiplicidad de parejas sexuales, generando vergüenza, culpa y estigma^17^. Además, la afiliación religiosa influye en la intensidad del estigma; así, los individuos de tradiciones conservadoras tenían más probabilidades de estigmatizar a las personas que viven con el VIH en comparación con otras religiones^18^.

Cabe destacar que todavía existe una asociación entre el VIH y el castigo divino, lo que intensifica las actitudes discriminatorias y compromete la adherencia al tratamiento. Así, la religión se configura como un factor con un doble efecto: puede reforzar los prejuicios cuando se guía por concepciones moralistas o contribuir a la reducción del estigma y al fortalecimiento del cuidado cuando se basa en discursos de inclusión y solidaridad^17^ ^-^ ^18^.

Este contexto también puede explicarse por el hecho de que las personas que no tienen una religión confían más en la medicina y así mantienen el tratamiento o vuelven a ella. Sin embargo, el deseo de colocar la propia vida bajo los principios de la religiosidad puede haberse originado en la creencia de que se podía salir de una condición de salud difícil, como el VIH^16^. 

Un estudio realizado en Zimbabwe con jóvenes que viven con el VIH mostró que poner sus vidas bajo la dirección de Dios a través de la religión estaba significativamente relacionado con un mayor fracaso del tratamiento^16^. 

Además, otra explicación es el hecho de que vivir con el VIH a menudo se asocia con comportamientos pecaminosos y los jóvenes que viven con el VIH, porque no eran considerados inocentes, tampoco eran dignos de protección (por las autoridades eclesiásticas)^16^, lo que hace que no tengan religión y busquen tratamiento.

De los factores analizados en cuanto a su influencia negativa en el retratamiento, la edad al inicio del seguimiento resultó ser un factor que disminuye las posibilidades de retorno. Sin embargo, es necesaria una verificación más profunda de los posibles determinantes adicionales, ya que el apoyo psicosocial, los familiares y las intervenciones educativas son importantes durante la juventud, lo que les permite tener una comprensión más holística de las conductas de adhesión al tratamiento^19^.

Además, los adolescentes y jóvenes que viven con el VIH son influenciados por condiciones externas estrechamente asociadas a ellos, como olvidos y problemas de salud mental, contribuyendo a una adhesión subóptima^20^.

Se observó que ser mujer disminuía las posibilidades de que los adolescentes y los pacientes jóvenes que vivían con el VIH volvieran al tratamiento. La diferencia de género puede estar relacionada con el hecho de que las mujeres acceden a más servicios de pruebas de VIH que los hombres, especialmente durante la atención prenatal^11^ ^,^ ^21^. Las mujeres que quedan embarazadas se adhieren al tratamiento porque quieren proteger al bebé se destacan. Un estudio realizado en el sur de Brasil identificó que el embarazo aumentaba las posibilidades de adhesión al tratamiento del VIH^22^ ^-^ ^23^.

En cuanto al hecho de que las mujeres no regresen al tratamiento, puede deberse a los desafíos de ser las principales cuidadoras de los niños y otros miembros de la familia, especialmente frente a la enfermedad. Y el hecho de que cuiden la salud de los familiares a menudo interfiere con la asistencia a las citas y la rutina con el TAR^11^ ^,^ ^21^ ^).^

La cuestión de que las mujeres no tengan un trabajo remunerado también puede influir en que no asistan a las consultas y sean retiradas del TAR^21^. Además, muchas mujeres tienen dificultad para revelar su estado serológico, por lo tanto, necesitan esconderse mientras toman el TAR y, por lo tanto, terminan olvidándose de tomar el medicamento^22^.

Cabe destacar que las desigualdades de género afectan directamente el acceso y la permanencia de las mujeres en el tratamiento. Además, se enfrentan a la sobrecarga de roles sociales, la dificultad de la negociación sexual, la dependencia económica y el estigma moral asociado a la seropositividad femenina; En consecuencia, se observan dificultades no solo en la adherencia inicial, sino también en el retratamiento. Por lo tanto, la feminización del VIH evidencia no solo el aumento de casos entre las mujeres, sino también las barreras simbólicas y estructurales que comprometen su continuidad en la atención^24^ ^-^ ^25^.

Vivir en un centro de tratamiento de drogas y/o detenidos también significa que existe una barrera para la conexión con el tratamiento del VIH debido a la falta de personas sin hogar. Por lo tanto, es necesario aumentar la participación en la atención entre las personas que viven con el VIH, revisando los modelos de prestación de servicios y reforzando el alcance de la prestación de atención tanto para abordar la vivienda como para la reducción de daños y el tratamiento de los trastornos por uso de sustancias, así como las intervenciones específicas de sexo y género^25^.

Un estudio realizado en Canadá muestra que la falta de vivienda se asoció con una reducción del 44% en las probabilidades de progresión general a través de la cascada de atención del VIH, con una reducción del 41% en las probabilidades de recibir TAR y una reducción del 54% en el mantenimiento de la adherencia y el logro de la supresión de la carga viral. Ante esto, se refuerza la necesidad de integración de servicios para abordar los desafíos interseccionales del VIH entre las poblaciones más vulnerables^26^.

Además, es importante incentivar la creación de una red de apoyo consolidada para las PVVIH para que puedan ser más perseverantes, seguras y adherirse mejor al tratamiento^27^.

La creación de apoyo social entre familiares y amigos, especialmente cuando los prejuicios y el estigma en la comunidad son fuertes, se convierte en una poderosa estrategia para la normalización social^28^ ^-^ ^29^. Por lo tanto, los profesionales de la salud deben incentivar a sus pacientes a crear nuevas relaciones sociales tanto con aquellos que aceptan el diagnóstico de VIH como con otras personas que viven con el VIH^28^. Así, durante la consulta de enfermería en el servicio especializado, el enfermero debe identificar y mapear la red de apoyo del paciente presente en el momento, con el propósito de desarrollar un plan de cuidados e incluirlo en él^27^.

El consumo de alcohol también fue un factor que dificultó el retorno a la atención del VIH, debido al deterioro de la memoria prospectiva y al comportamiento interactivo de las creencias de toxicidad/evitación debido al deterioro de la enfermedad física y el comportamiento social. Por lo tanto, se desarrolla dependencia al alcohol, cuya abstinencia causa efectos adversos y, por lo tanto, puede llevar a la no adhesión intencional y no intencional^30^ ^-^ ^31^.

Otra situación que puede interferir con el retratamiento es la estigmatización del consumo de alcohol entre las personas que viven con el VIH que están usando TAR, es decir, la sociedad tiene la percepción de que el consumo de alcohol es vergonzoso e irresponsable debido a los comportamientos del individuo; Así, los efectos negativos del consumo de alcohol en la adhesión al tratamiento se agravan, comprometiendo la eficacia del tratamiento y la progresión de la enfermedad^32^ ^-^ ^33^.

Así, los servicios de salud deben ser conscientes del abuso de alcohol entre las personas que viven con el VIH, especialmente los adolescentes y jóvenes, que, por las características de su propia edad, ya están sujetos a conductas de riesgo para la salud. Dicho esto, es necesario que la atención sea integrada y que pueda rastrear, tratar y monitorear el abuso de alcohol y el VIH, reduciendo así la brecha en el tratamiento y los malos resultados del uso de esta sustancia en todas las etapas de la atención del VIH^32^ ^-^ ^33^.

El diagnóstico en el CTA también influye en el regreso de la atención del VIH y, aunque el servicio respectivo en el que se llevó a cabo este estudio cuenta con asesoramiento, los propios adolescentes y jóvenes tienen que lidiar con el estigma que compromete la capacidad de tomar el TAR abiertamente y sin miedo. Y con una adherencia comprometida, la carga viral no suprimida es casi inevitable. Por lo tanto, la consejería debe resaltar la naturaleza crónica de la enfermedad y la necesidad de tratar con TAR para toda la vida^34^.

Un estudio realizado en Uganda muestra que una buena adherencia al TAR es crucial para la supresión de la CV, pero muchos jóvenes no son conscientes de este hecho. Además, los adolescentes que nunca lograron una CV suprimida después de iniciar el tratamiento expresaron preocupación por no conocer la justificación para tomar píldoras periódicamente^34^; por lo tanto, este contexto también puede influir en el retratamiento.

La falta de consultas antes del abandono es un factor que reduce las posibilidades de retratamiento entre adolescentes y jóvenes que viven con VIH, y los pacientes que presentan este comportamiento son considerados desviados o no conformes al tratamiento por parte de los profesionales de la salud y del sistema general de salud^21^.

Los adolescentes y jóvenes que viven con el VIH necesitan un tratamiento contra el VIH de por vida, y las consultas médicas, las extracciones regulares de sangre y los medicamentos desagradables son cruciales^35^. Y además de esto, todavía lidian con contextos físicos, emocionales y sociales que afectan la toma de decisiones y comportamientos relacionados con el seguimiento y la adherencia al tratamiento. Además, la pérdida de seguimiento también empeora desde el inicio de la adolescencia hasta el final de la juventud/adultez temprana, posiblemente debido a la menor participación de los cuidadores y al aumento de la responsabilidad y autonomía^35^.

Una estrategia para reducir las citas perdidas y los retiros del TAR es la prescripción por varios meses (3 o 6 meses) o el uso de nuevas tecnologías, como el TAR inyectable de acción prolongada, que por lo tanto pueden facilitar a los jóvenes conciliar las prioridades de atención al VIH y la vida fuera del servicio especializado^21^.

Además, las estrategias adicionales para mejorar la resiliencia y el alcance de los pacientes después de cualquier brecha de tratamiento pueden facilitar el retorno, así como aumentar la conciencia de los gestores y profesionales de la salud para reconocer los esfuerzos de los pacientes para permanecer en el seguimiento^21^ ^,^ ^36^.

En cuanto a las limitaciones del estudio, se señala el uso exclusivo de datos secundarios de las historias clínicas de adolescentes y jóvenes, que pueden presentar información incompleta y, por lo tanto, sesgo de interpretación.

Las implicaciones para el avance del conocimiento científico en el área de la salud y la enfermería están relacionadas con el desarrollo de un estudio que responda a un vacío de conocimiento dirigido a este público. Los resultados permiten a los enfermeros y otros profesionales de la salud desarrollar un plan de cuidados basado en las necesidades de los pacientes adolescentes y jóvenes con el fin de prevenir el abandono permanente del tratamiento. Se sugieren estudios futuros sobre la reincorporación, es decir, el regreso al tratamiento de adolescentes y jóvenes que viven con el VIH, centrándose en las medidas de estos predictores para investigar sus mecanismos de efecto causal en el retratamiento.

## Conclusión 

El retorno al tratamiento de adolescentes y jóvenes que viven con VIH tiene determinantes multifactoriales asociados al perfil sociodemográfico, conductual y clínico. Se observó que no tener práctica religiosa interfiere con el retratamiento y los factores asociados negativamente con el retorno de este público al servicio fueron ser mujeres, consumir alcohol, vivir en una institución, diagnóstico en la CTA y tener ausentismo en las consultas antes de la pérdida de seguimiento. Estos resultados proporcionan información sobre la necesidad de revisar las prácticas de salud para prevenir la deserción permanente y promover la retención de adolescentes y jóvenes en el tratamiento del VIH. Además, se destaca la necesidad de acciones prácticas, como el fortalecimiento de las políticas públicas externas dirigidas a la vivienda, una mayor inclusión de las mujeres (no solo las embarazadas) y la reducción de los prejuicios en las estrategias de prevención, tratamiento y atención del VIH entre adolescentes y jóvenes.

## Data Availability

Todos los datos generados o analizados durante este estudio están incluidos en este artículo publicado.
